# Facile transmetallation of [Sb^III^(DOTA)]^−^ renders it unsuitable for medical applications†

**DOI:** 10.1039/d2ra00642a

**Published:** 2022-02-16

**Authors:** Catherine Chen, Charlotte Sommer, Helge Thisgaard, Vickie McKee, Christine J. McKenzie

**Affiliations:** Department of Physics, Chemistry and Pharmacy, University of Southern Denmark Campusvej 55 5230 Odense M Denmark mckenzie@sdu.dk +45 6615 8760 +45 6550 2518; Department of Nuclear Medicine, Odense University Hospital Odense Denmark; Department of Clinical Research, University of Southern Denmark Odense Denmark; School of Chemical Sciences, Dublin City University Glasnevin Dublin 9 Ireland

## Abstract

The antimony(iii) complex of 1,4,7,10-tetraazacyclododecane-1,4,7,10-tetraacetate (DOTA) has been prepared and its exceptionally low stability observed. The Sb(iii) ion in Na[Sb(DOTA)]·4H_2_O shows an approximately square antiprismatic coordination geometry that is close to superimposable to the Bi(iii) geometry in [Bi(DOTA)]^−^ in two phases containing this anion, Na[Bi(DOTA)]·4H_2_O, [H_3_O][Bi(DOTA)]·H_2_O for which structures are also described. Interestingly, DOTA itself in [(H_6_DOTA)]Cl_2_·4H_2_O·DMSO shows the same orientation of the N_4_O_4_ metal binding cavity reflecting the limited flexibility of DOTA in an octadentate coordination mode. In 8-coordinate complexes it can however accommodate M(iii) ions with *r*_ion_ spanning a relatively wide range from 87 pm (Sc(iii)) to 117 pm (Bi(iii)). The larger Bi^3+^ ion appears to be the best metal–ligand size match since [Bi(DOTA)]^−^ is associated with greater complex stability. In the solution state, [Sb(DOTA)]^−^ is extremely susceptible to transmetallation by trivalent ions (Sc(iii), Y(iii), Bi(iii)) and, significantly, even by biologically important divalent metal ions (Mg(ii), Ca(ii), Zn(ii)). In all cases just one equivalent is enough to displace most of the Sb(iii). [Sb(DOTA)]^−^ is resistant to hydrolysis; however, since biologically more abundant metal ions easily substitute the antimony, DOTA complexes will not be suitable for deployment for the delivery of the, so far unexploited, theranostic isotope pair ^119^Sb and ^117^Sb.

## Abstract 2

The antimony(iii) complex of 1,4,7,10-tetraazacyclododecane-1,4,7,10-tetraacetate (DOTA) has been prepared and its exceptionally low stability observed.

## Graphical abstract

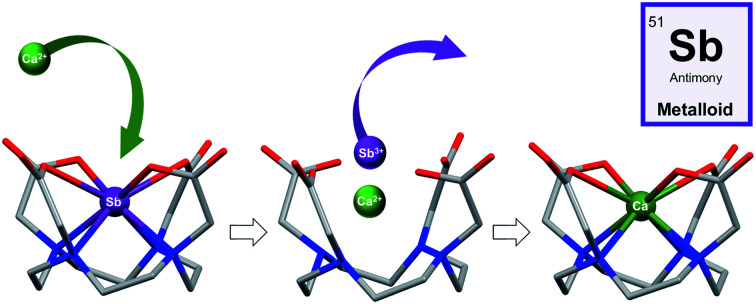

## Introduction

**Fig. 1:**
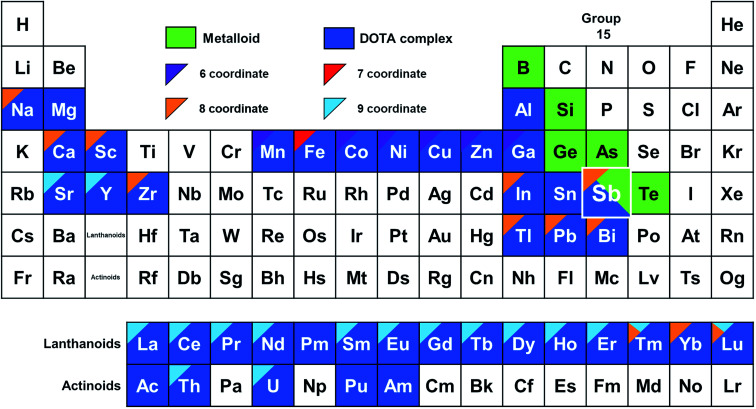
A DOTA periodic table. Elements for which DOTA complexes have been characterized are colored dark blue.^16,19–62^ The color of the corners denote coordination number determined from crystal structures according to the legend in the figure.^16,19–48,62^ Metalloid elements are colored green. The structural information for Sb is from the present work and illustrates this is the first DOTA complex of a metalloid.

The main group pnictogen, arsenic, antimony, and bismuth compounds have a long history in medicinal use as treatments for syphilis, skin lesions, leishmaniasis, and gastrointestinal disorders with varying degrees of success.^1–3^ Renewed interest as anti-cancer, anti-microbial, and anti-parasitic treatments has spurred the search for better chelating ligands for these elements with the aim of improving efficacy through targeted therapies.^4–8^ DOTA (1,4,7,10-tetraazacyclododecane-1,4,7,10-tetraacetate) is a chelating ligand prolifically used for the medical delivery of metal ions, and the ^213^Bi (α decay, 45.59 min half-life) complex has been applied for Targeted Alpha Therapy (TAT) in the treatment of cancers including recurrent glioblastomas.^9,10^ The ability of DOTA to form stable complexes in water with a wide range of metal ions with ionic radii from 75–117 pm (Fig. 1) means that it is routinely used in magnetic and nuclear medical diagnostics (PET, SPECT, MRI) and radiotherapeutic applications. This tetraglycyl appended cyclam-based macrocycle supports 6–9 coordination due to the bifunctional glycyl arms which coordinate, or not, according to the preferences of a particular metal ion. Nine-coordination is common for the larger metal ions and this is achieved with a co-ligand, usually water (an essential feature for MRI). DOTA has therefore the status of chelator of choice, forming exceptionally stable complexes with many metallic radiopharmaceuticals and the scaffold is well developed with respect to protocols for conjugating with targeting moieties.^11–13^ We are interested in the implementation of antimony in nuclear medicine. The ^117^Sb (β^+^ decay, *T*_½_ = 2.8 h) and ^119^Sb (electron capture, *T*_½_ 38.19 h half-life) isotopes can be used for Single-Photon Emission Computed Tomography (SPECT) imaging and radioisotope therapy (RIT) respectively. Hence these two isotopes of antimony furnish a so far unexploited theranostic isotope pair.^14^ Since DOTA forms a stable complex with bismuth,^15,16^ and has been claimed to be a potential ligand for arsenic,^17^ the exploration of its ability to complex the pnictogen antimony seemed warranted in the search for suitable antimony-binding ligands for medical use.

Very recently, Tóth-Molnár *et al.* reported that complex formation between DOTA and Sb(iii) (from Sb_2_O_3_, antimony tartrate or SbCl_3_) fails under aqueous reaction conditions. This was due to rapid formation of insoluble precipitates from Sb(iii) hydrolysis.^18^ While this reactivity is a significant synthetic challenge, we have found that the synthesis of [Sb(DOTA)]^−^ is possible in ethanol and this has allowed for its isolation, structural characterization and studies of stability. Notably, once formed [Sb(DOTA)]^−^ is stable towards hydrolysis. We find however that other metal ions, significantly the biological ions (Mg^2+^, Ca^2+^, Zn^2+^) that are present in excess in cells compared to any therapeutic complex, can easily substitute Sb^3+^ under aqueous conditions. We can therefore conclude that this reactivity will preclude the use of DOTA, an otherwise ubiquitously used chelator for medical applications, for medical delivery of antimony.

## Results and discussion

### Syntheses of DOTA complexes of pnictogen metal(iii) ions

**Fig. 2:**
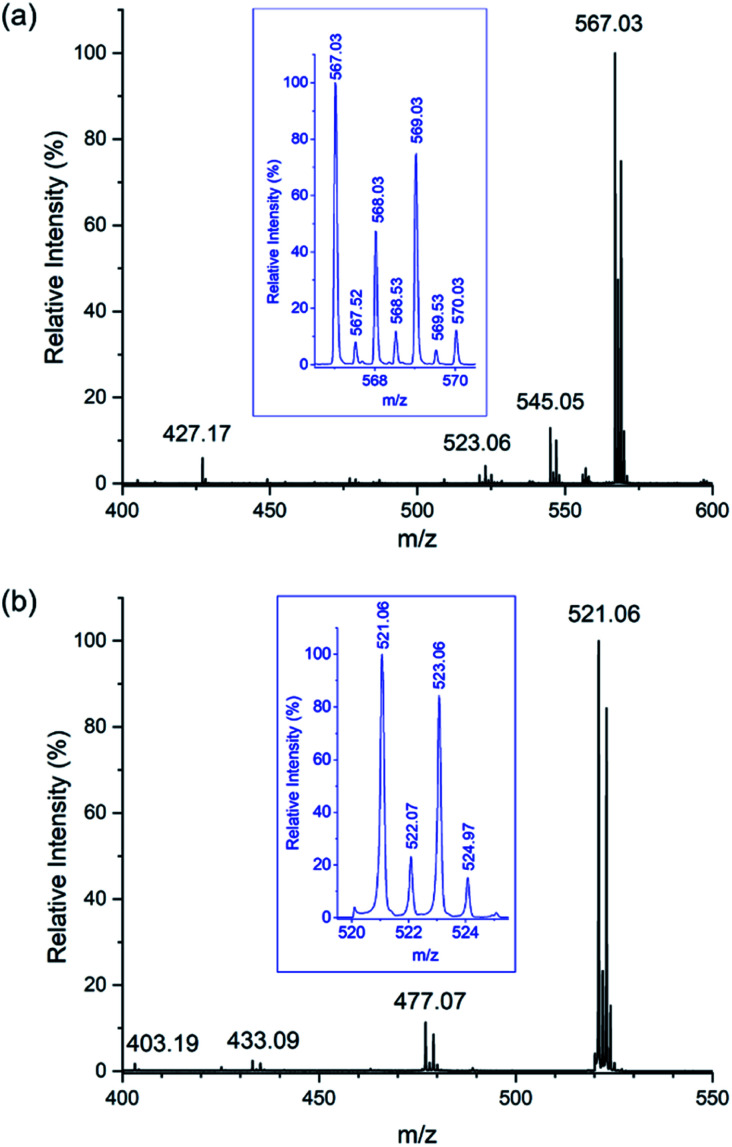
ESI mass spectra of Na[Sb(DOTA)]·4H_2_O in water : acetonitrile (1 : 50) recorded in recorded in; (a) positive mode showing peaks corresponding to [NaH_4_DOTA]^+^ (*m*/*z* 427.17), [H_2_Sb(DOTA)]^+^ (*m*/*z* 523.06), [NaHSb(DOTA)]^+^ (*m*/*z* 545.05), and [Na_2_Sb(DOTA)]^+^ + {[Na_2_Sb(DOTA)]_2_}^2+^ (*m*/*z* 567.03) (expanded in inset with deconvolution shown in Fig. S1†); (b) negative mode showing peaks corresponding to [H_3_DOTA]^−^ (*m*/*z* 403.19), {[Sb(DOTA)]-2CO_2_}^−^ (*m*/*z* 433.09), {[Sb(DOTA)]-CO_2_}^−^ (*m*/*z* 477.07), and [Sb(DOTA)]^−^ (*m*/*z* 521.06) (expanded in inset).

DOTA complexes are typically easily prepared in water using metal salts, sometimes with pH adjustment.^15,19,23,35,63–67^ However, salts of the trivalent pnictogens antimony and bismuth undergo facile hydrolysis, forming highly insoluble oxy-hydroxide species and this reactivity can hamper the syntheses of their complexes in water.^68–70^ In the particular case of Sb(iii), dissolution of SbCl_3_ (even in the presence of small amounts of water) produces insoluble oxy-hydroxides immediately. The hydrolysis reactions are irreversible for both Bi(iii) and Sb(iii) in the presence of H_4_DOTA over pH range 3–10. Hydrolysis could however be circumvented by employing Na_4_(DOTA)^16^ in a complexation reaction using SbCl_3_ in absolute ethanol. Surprisingly, recrystallisation of the product in water (pH 7.0) was possible without formation of insoluble oxy-hydroxides, to yield the tetrahydrate sodium salt, Na[Sb(DOTA)]·4H_2_O. The formation of insoluble oxy-hydroxides in water is less of a synthetic challenge in the preparation of Na[Bi(DOTA)]·4H_2_O and [H_3_O][Bi(DOTA)]·H_2_O with the pH determining which of these phases was obtained (pH 7 and 2 respectively). Once formed, [Bi(DOTA)]^−^ is stable to hydrolysis, as attested by its clinical use. Ions pertaining to [Sb(DOTA)]^−^ are observed in the ESI mass spectra for Na[Sb(DOTA)]·4H_2_O in both positive and negative mode (Fig. 2) and corresponding spectra for the Bi(iii) and Y(iii) complexes can be found in ESI (Fig. S2 and S3†). Stability constants of 30.3,^16^ 24.4 ^71^ and 27.0 ^72^ have been reported for [Bi(DOTA)]^−^, [Y(DOTA)(H_2_O)]^−^ and [Sc(DOTA)]^−^, respectively. High *in vivo* stability, of this order of magnitude, is a prerequisite for using chelated isotopes in diagnostic medical imaging and therapy.

### X-ray crystal structures of [H_6_DOTA]Cl_2_·4H_2_O·DMSO, Na[M(DOTA)]·4H_2_O M = Sb, Bi and [H_3_O][Bi(DOTA)]·H_2_O

**Fig. 3:**
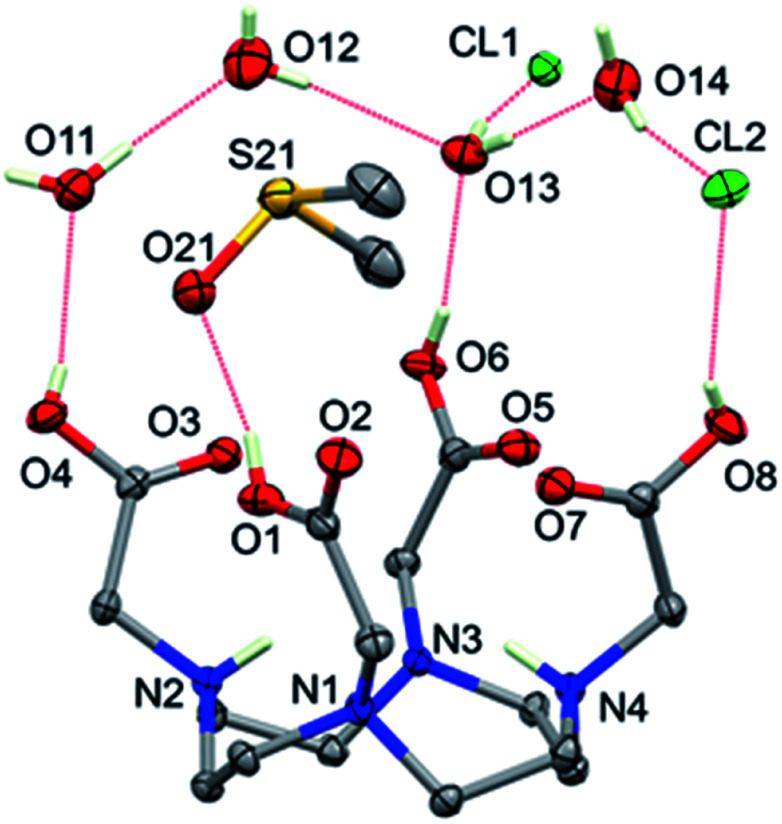
Formula unit of [H_6_DOTA]Cl_2_·4H_2_O·DMSO, showing 50% probability ellipsoids. Dotted red lines indicate hydrogen bonds. Hydrogen atoms bonded to carbon and a small disorder of O11 and Cl2 are omitted for clarity.

H_4_DOTA was recrystallized as [H_6_DOTA]Cl_2_·4H_2_O·DMSO from hydrochloric acid (0.1 M) : DMSO (1 : 1). The formula unit (also the asymmetric unit) is shown in Fig. 3; two of the amine nitrogen atoms and all four carboxylic acid groups in the [H_6_DOTA]^2+^ unit are protonated. The conformation of the [H_6_DOTA]^2+^ ion is very similar to that reported for [H_6_DOTA]Cl_2_·5H_2_O ^73^ and to the neutral unit in [H_4_DOTA]·2H_2_O;^74^ these three structures are compared in Fig. S18.† The DMSO solvate lies over the center of the [H_6_DOTA]^2+^ ion and is hydrogen bonded to a carboxylic acid (O1). The other three acid groups are also hydrogen bonded; two of them to water molecules and one to a chloride anion. Neighboring [H_6_DOTA]^2+^ ions are linked *via* a C–H⋯O hydrogen-bonding network involving the water molecules and chloride anions (Fig. S16 and S17 and Table S1†).

**Fig. 4:**
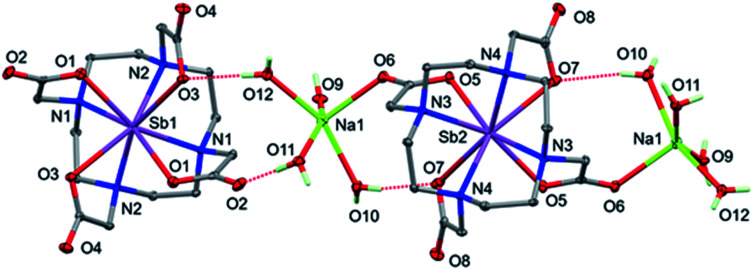
Perspective view of Na[Sb(DOTA)]·4H_2_O, showing [Sb(DOTA)]^−^ anions and [(Na(H_2_O)_4_)_2_Sb(DOTA)]^+^ cations. Non-hydrogen atoms shown with 50% probability ellipsoids, only the H atoms of the water molecules are included, and OH⋯O H-bonds are shown dotted.

The structures of Na[Sb(DOTA)]·4H_2_O and Na[Bi(DOTA)]·4H_2_O are isomorphous and both were solved in space group *P*2/*c*. The unit cell dimensions are the same as those reported previously for Na[Bi(DOTA)]·4H_2_O ^16^ but the published structure was solved in *C*2/*c* with some disorder affecting the sodium ion and water molecules; our data sets can be also solved in *C*2/*c* to replicate the published result. However, examination of the data showed that the data are not, in fact C-centered. Since the refinement in *P*2/*c* shows no disorder, we conclude that this is the correct choice and that the apparent centering is a consequence of the majority of the electron density being on the heavy atoms which have higher symmetry than the overall structure. The asymmetric unit contains two independent half [M(DOTA)]^−^ anions (where M = Sb or Bi), with the metal ions on 2-fold axes with approximately square antiprismatic coordination geometry. One [M(DOTA)]^−^ anion is directly coordinated to the sodium counterions and each sodium ion is also coordinated to four water molecules (Fig. 4). Fig. S19 and S20† show the resulting polymeric chain of alternating [Sb(DOTA)]^−^ anions and [(Na(H_2_O)_4_)_2_Sb(DOTA)]^+^ cations. Very similar figures for the bismuth analogue are shown in Fig. S21 and S22,† selected bond lengths are listed in Table 1.

**Table 1:** Selected bond lengths (Å) for Na[Sb(DOTA)]·4H_2_O and Na[Bi(DOTA)]·4H_2_O

Na[Sb(DOTA)]·4H_2_O		Na[Bi(DOTA)]·4H_2_O	
Sb1–O1	2.5011 (10)	Bi1–O1	2.4993 (11)
Sb1–O3	2.5971 (10)	Bi1–O3	2.5715 (11)
Sb1–N1	2.4563 (11)	Bi1–N1	2.5288 (13)
Sb1–N2	2.4453 (11)	Bi1–N2	2.5170 (12)
Sb2–O5	2.5146 (10)	Bi2–O5	2.5012 (11)
Sb2–O7	2.6929 (11)	Bi2–O7	2.6336 (11)
Sb2–N3	2.4421 (12)	Bi2–N3	2.5256 (13)
Sb2–N4	2.4277 (11)	Bi2–N4	2.5034 (12)
Na1–O6	2.3483 (12)	Na1–O6	2.3531 (13)
Na1–O9	2.3074 (13)	Na1–O9	2.3093 (13)
Na1–O10	2.3653 (12)	Na1–O10	2.3705 (13)
Na1–O11	2.6005 (13)	Na1–O11	2.6204 (14)
Na1–O12	2.3341 (12)	Na1–O12	2.3383 (13)

Although all the [M(DOTA)]^−^ units have approximate square prismatic geometry, there are significant differences in the details between Sb and Bi complexes (Tables 1 and S2†). The M–N bonds within each ion are similar but not identical, and the Sb–N distances are shorter than those for Bi–N, though not by as much as might be expected from the differences in their ionic radii (*ca*. 0.3 Å). The two independent M–O distances are distinctly different within each [M(DOTA)]^−^ ion and, strikingly, the Bi–O bonds are *shorter* than those to the ostensibly smaller Sb^3+^ ion. It is also relevant to compare the structure of the [Sc(DOTA)]^−^ anion,^62^ because Sc(iii) and Sb(iii) can be expected to show very similar crystal radii for the 8 coordination (*ca.* 87 pm).‡‡The 87 pm quoted is the Shannon Prewitt radius for 8 coordinated Sc(iii). We were unable to find the corresponding radius for 8 coordinated Sb(iii). The radii for 6 coordinated Sc(iii) and Sb(iii) are however very close at 75 pm and 76 pm respectively. We have therefore assumed that the 8 coordinated radius for Sb(iii) will also be close to 87 pm. The M–N distances are very similar (*ca.* 2.4 Å) for the structures of [M(DOTA)]^−^ M = Sc, Sb, but the Sc–O bonds (2.223(2) Å) are significantly shorter than the Sb–O bonds that range between 2.501(1) Å and 2.693(1) Å (see overlay in ESI Fig. S26†). When similar ionic radii for 8-coordinated Sc^3+^ and Sb^3+^ are expected, why are these anions not isostructural? Two important effects may be at play; (i) the presence of an active lone pair on the main group Sb(iii), potentially this might be located in the center of the face defined by the 4 O atoms with the consequence that the metal is nearer to the N donors, and (ii) hard/soft acid/base (HSAB) effects: despite the same size and charge Sb(iii) is a softer ion than the more oxophilic Sc(iii).

**Fig. 5:**
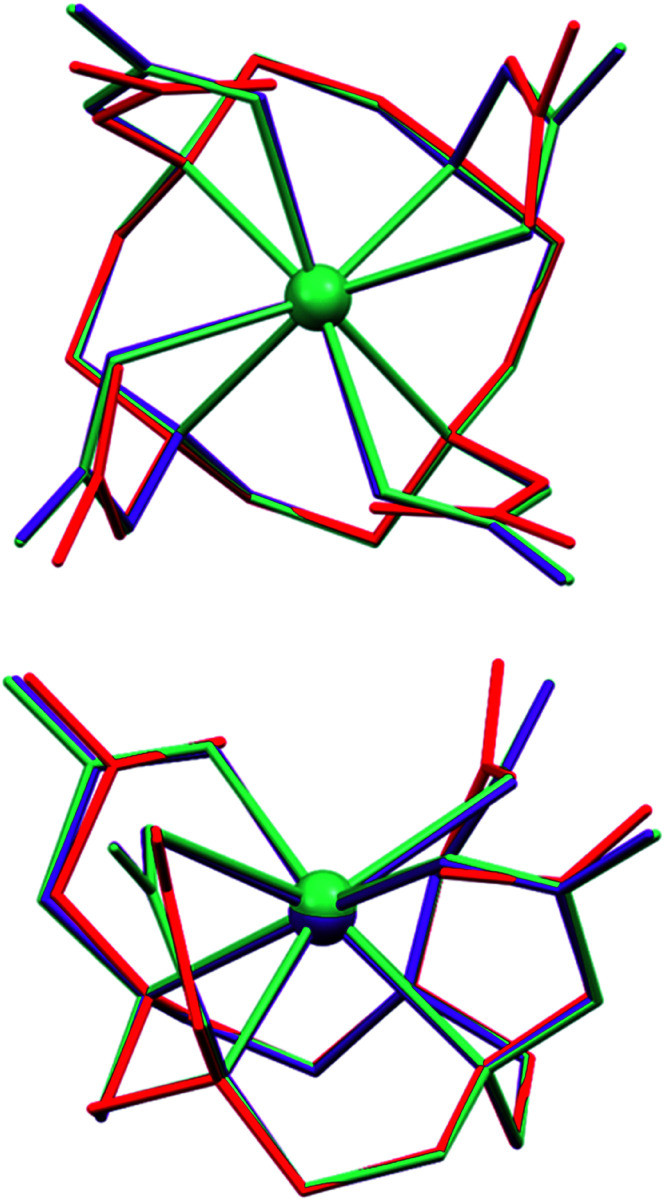
Overlays of the [Sb(DOTA)]^−^ (purple) and [Bi(DOTA)]^−^ (green) anions with the [H_6_DOTA]^2+^ ion from [H_6_DOTA]Cl_2_·4H_2_O·DMSO structure (red).

Some of this difference also reflects the restricted flexibility of the octadentate DOTA unit, essentially limited to rotation of the carboxylate groups. If the antimony sits closer to the plane of the nitrogen donors, it must have longer bonds to O donors as the carboxylates cannot contract towards the metal ion without inducing strain in the DOTA framework. The mean Sb out-of-plane distances are 1.336 for the N4 and 1.133 Å for the O4 donor atom planes; the corresponding values for [Bi(DOTA)]^−^ are 1.428 and 1.109 Å, and for [Sc(DOTA)]^−^ 1.336 and 0.989 Å,^24^ respectively. The restricted flexibility of the cavity is illustrated by the close match in the overlays of the free H_4_DOTA ligand^74^ with the [Sb(DOTA)]^−^ and [Bi(DOTA)]^−^ ions (Fig. 5).

**Fig. 6:**
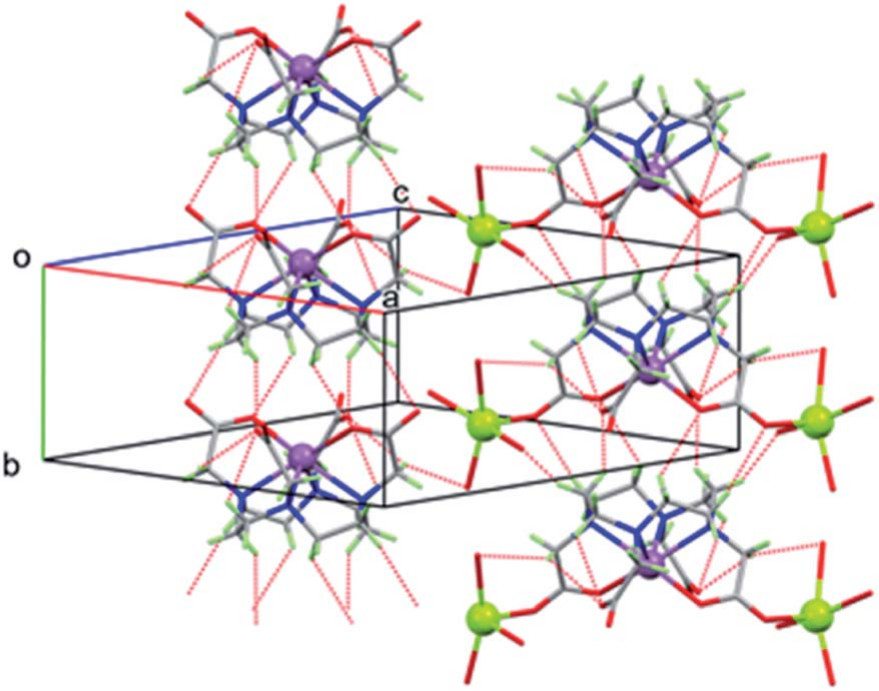
Packing diagram of Na[Sb(DOTA)]·4H_2_O showing [Sb(DOTA)]^−^ units stacking parallel to the *b* axis *via* CH⋯O H bonds (dotted lines), H atoms of water molecules omitted for clarity.

Unexpectedly, the geometry at the sodium ion is approximately square pyramidal; there is a sixth interaction with O2 of the adjacent [M(DOTA)]^−^ unit but the M–O2 distances are very long (3.2695(13) and 3.1994(14) Å for Sb and Bi, respectively). Additionally, the bond to one of the coordinated water molecules (O11) is significantly longer than the others in both structures (Table 1). Each of the coordinated water molecules makes two hydrogen bonds to carboxylate oxygen atoms, with the exception of O9, which makes one hydrogen bond to carboxylate and the other to water (O11). The OH⋯O hydrogen bonds are listed in Table S3† and shown in Fig. 4. Given the irregular geometry about the sodium ion, it seems likely that there are interactions competing with these to control the crystal packing. The most likely candidate is the extensive set of intermolecular CH⋯O interactions (Table S3,†Fig. 6 and S21†). These generate head-to-tail stacks of [Sb(DOTA)]^−^ units running parallel to the *b* axis as well as a smaller number of links between the columns. Similar stacking motifs are present in the structure of [H_4_DOTA]·H_2_O ^74^ as well as several other 8- or 6-coordinate DOTA^4−^ complexes.^24,25,41,44–46,75^

**Fig. 7:**
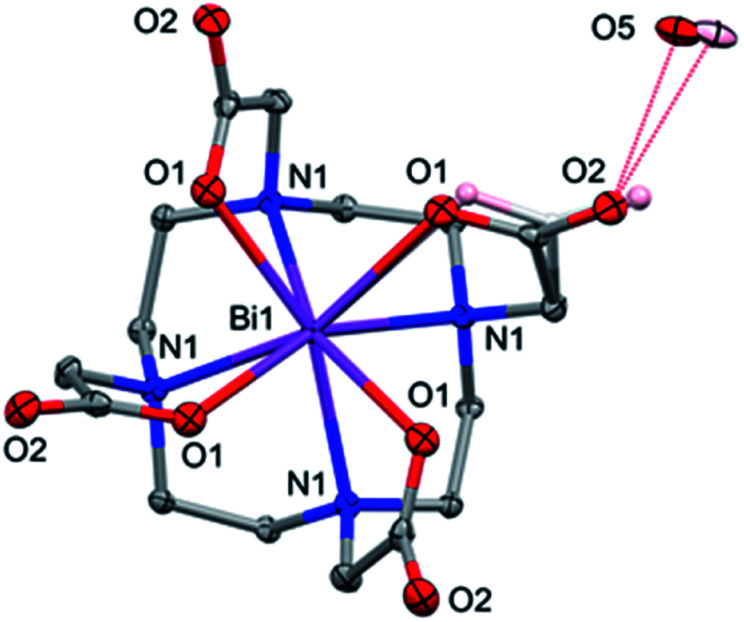
[H_3_O][Bi(DOTA)]·H_2_O showing the disorder for one of the four equivalent carboxylate groups and the H_3_O^+^/H_2_O (pink and light grey). Atoms are drawn with 50% probability ellipsoids except for the minor component of the disordered carboxylate.

Powder X-ray diffraction indicated that a second phase of a bismuth complex forms under more acidic conditions (Fig. S14†). Single crystals of this phase, [H_3_O][Bi(DOTA)]·H_2_O, were obtained by diffusion of ethanol into a concentrated solution of Na[Bi(DOTA)]·4H_2_O in 0.15 M aq. nitric acid. The [Bi(DOTA])^−^ anion has the expected structure with the Bi ion lying on a 4-fold axis, so that the four pendant arms are equivalent. There is some disorder of the carboxylate group which refined to approximately 77% occupation of the major orientation and 23% for the minor component, which was refined isotropically. A disordered H_3_O^+^/H_2_O counter-cation/solvate lies close to a rotation-inversion axis (the coincident 2-fold rotation axis is more obvious) and is involved in hydrogen bonding to the carboxylate oxygen atoms (Fig. 7).

Assuming the bismuth is in the +3 state, charge balance requires one of the following: (i) one carboxylate group is not deprotonated; (ii) there is one H_3_O^+^ per [Bi(DOTA])^−^ ion; or (iii) there is a Na^+^ ion present, as in the Na[Bi(DOTA)]·4H_2_O complex. Given the 4-fold symmetry and the disorder, it is not possible to detect whether there is a proton on the [Bi(DOTA)]^−^ unit (though this seems not unreasonable at pH 2). The fit to the data is however better for a H_3_O^+^/H_2_O unit than for a model containing Na^+^ as counter ion (further details in the ESI†). So, on balance, [H_3_O][Bi(DOTA)]·H_2_O seems the most appropriate model, with the caveat that the location of the acidic proton is not unambiguously determined.

### Competitive metal binding

The affinity of selected biological and medical metal ions for DOTA at was probed using Electrospray Ionization (ESI) Mass spectrometry. M^2+^ (Ca, Mg and Zn) and M^3+^ (Sb, Bi and Y) ions (0.15 mM each) and an excess of H_4_DOTA (1 mM) were mixed in water at pH 7.0 and allowed to stand for at least 2 h. The mixture was then diluted 10-fold with MeCN and the mass spectra recorded. The negative ion spectra show an ion assignable to [H_3_DOTA]^−^ as the most intense signal and contains ions assignable to [M^III^(DOTA)]^−^ M = Y and Bi complexes with 20% and 15% relative intensity. Weaker ions assigned to [HZn(DOTA)]^−^ and [Sb^III^(DOTA)]^−^ appear with low intensity (5%). The positive ion spectra revealed ions assignable to protonated complexes for all the metal ions present, for example [H_2_Y(DOTA)]^+^ (77.8%, *m*/*z* 491.06 calc. 491.08), [H_3_Zn(DOTA)]^+^ (58.0%, *m*/*z* 467.09 calc. 467.11) and [H_2_Bi(DOTA)]^+^ (21.2%, *m*/*z* 611.13 calc. 611.15), which were observed with higher relative intensities compared to those for [H_3_Ca(DOTA)]^+^ (9.16%, *m*/*z* 443.12 calc. 443.14) and [H_2_Sb(DOTA)]^+^ (10.7%, *m*/*z* 523.05 calc. 523.07). The conclusion that can be made from the series of experiments is that the formation of Y^3+^, Bi^3+^ and Zn^2+^ complexes is highly preferred over those for the Ca^2+^ and Sb^3+^ ions (Fig. S4†).

**Fig. 8:**
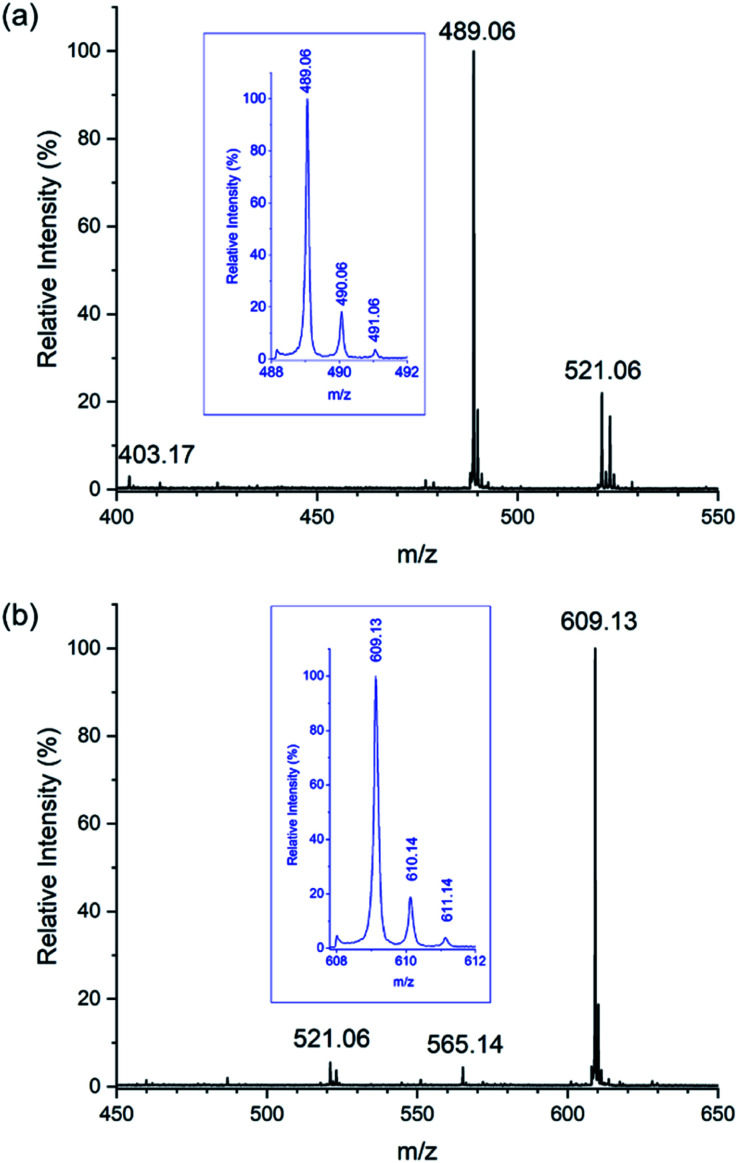
ESI mass spectra recorded in negative mode of samples containing acetonitrile : water (9 : 1). Samples were diluted from aqueous solutions containing Na[Sb(DOTA)]·4H_2_O (0.5 mM) incubated at 298 K with (a) Y(NO_3_)_3_·6H_2_O (0.5 mM) showing almost complete replacement of the Sb^3+^ with Y^3+^ showing peaks corresponding to [Y(DOTA)]^−^ (*m*/*z* 489.06), [Sb(DOTA)]^−^ (*m*/*z* 521.06), and [H_3_DOTA]^−^ (*m*/*z* 403.17), and with (b) Bi(NO_3_)_3_·5H_2_O (0.5 mM), showing almost complete replacement of Sb^3+^ with Bi^3+^ with peaks corresponding to [Bi(DOTA)]^−^ (*m*/*z* 609.13), {[Bi(DOTA)]–CO_2_}^−^ (*m*/*z* 565.14), and [Sb(DOTA)]^−^ (*m*/*z* 521.06).

**Fig. 9:**
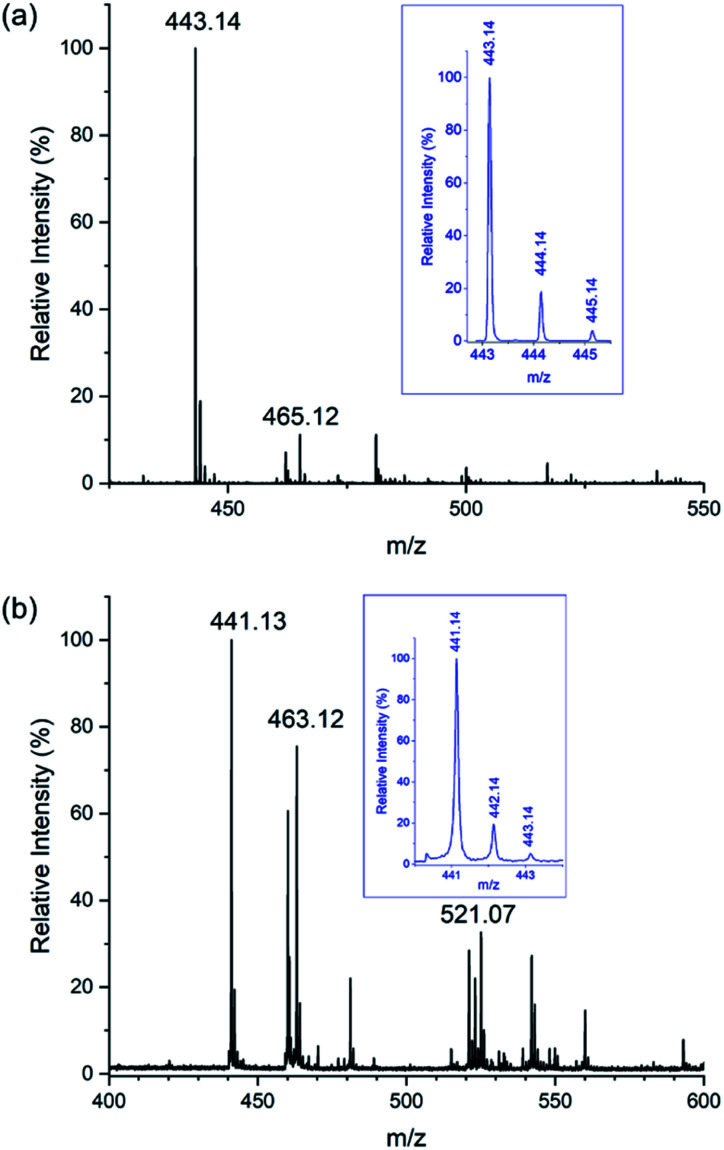
ESI mass spectra recorded in negative mode of samples containing acetonitrile: water (9 : 1). Samples were diluted from aqueous solutions containing Na[Sb(DOTA)]·4H_2_O (0.5 mM) incubated with Ca(NO_3_)_2_·4H_2_O (1 mM) at 298 K showing the replacement of Sb^3+^ with Ca^2+^ recorded in; (a) positive mode with peaks corresponding to [H_3_Ca(DOTA)]^+^ (*m*/*z* 443.14) (expanded in inset) and [NaH_2_Ca(DOTA)]^+^ (*m*/*z* 465.12) and (b) negative mode with peaks corresponding to [HCa(DOTA)]^−^ (*m*/*z* 441.14) (expanded in inset), [NaCa(DOTA)]^−^ (*m*/*z* 463.12), and [Sb(DOTA)]^−^ (*m*/*z* 521.07).

In competition experiments, aqueous solutions of Na[Sb(DOTA)]·4H_2_O (1 mM) were mixed with one or two equivalents of M^2+^ (Ca, Mg and Zn) and M^3+^ (Sc, Y and Bi) ions (1 mM). The mass spectra of these solutions shows that Sb^3+^ undergoes facile replacement by all these metal ions. Addition of 1 molar equivalent of Bi(NO_3_)_3_·5H_2_O, Y(NO_3_)_3_·6H_2_O, or Sc(ClO_4_)_3_ results in near stoichiometric replacement of the Sb^3+^; 2 molar equivalents results in the complete replacement according to the MS spectra (Fig. 8, S5 and S6†). Incubation of aqueous solutions of [Sb(DOTA)]^−^ with two equivalents of Ca(NO_3_)_2_·4H_2_O, MgCl_2_·6H_2_O and ZnCl_2_ also result in the replacement of Sb^3+^ and consequent formation of [M^II^(DOTA)]^2−^ ions. Not unexpectedly, this occurs to a lesser extent compared with the M^3+^ ions, with the following trends: Ca^2+^ > Zn^2+^ > Mg^2+^ (*ca.* 65%, 55% and 38% substitution respectively, Fig. 9, S7–S9†). These observations follow more or less the trends expected from the stability constants, log *K*, for the formation of [M^II^(DOTA)]^2−^ and [M^III^(DOTA)]^−^ complexes: Bi^3+^ = 30.3 ^16^ > Sc^3+^ = 27.0 ^72^ > Y^3+^ = 24.4 ^71^ > Zn^2+^ = 18.7 ^61^ > Ca^2+^ = 16.4 ^61^ > Mg^2+^ = 11.2.^61^ These have been measured using UV-Visible spectrophotometry,^16,76,77^ HPLC chromatography^72^ or isotachophoresis^71^ in aqueous solutions. The conspicuous absence of stability constants for the antimony complex in the literature is due to the rapid hydrolysis of Sb^3+^ and Sb^5+^ ions in aqueous solutions.^78^ Reported stability constants for Sb^3+^ complexes are often not obtained from direct measurements, but derived from extrapolations using literature data and studies conducted in highly acidic media, and applied to complex models.^18,78^ Without the ability to derive stability constants using direct measurements, the use of these extrapolated values are subject to conjecture especially for application to biological systems.

The results suggest that the stability of the Sb^3+^ complex is significantly lower than corresponding DOTA complexes with trivalent metals, Bi^3+^, Sc^3+^, and Y^3+^ and significantly, even the biologically relevant divalent metals, Ca^2+^, Mg^2+^, and Zn^2+^. The physiological concentrations of Ca^2+^, Mg^2+^, and Zn^2+^^79,80^ are greater by at least 4, 3 and 2 orders of magnitude respectively compared with clinical radiopharmaceutical dosages.^81–83^ Even though we have now isolated and characterized the elusive [Sb(DOTA)]^−^ in the solid state, our results show that antimony complexes of DOTA can be expected to undergo rapid transmetallations *in vivo*. By contrast the Y^3+^, and Bi^3+^ ions in aqueous [M^III^(DOTA)]^−^ complexes do not undergo substitution by any of the M^2+^ and M^3+^ ions explored (Fig. S10–S13†).

## Conclusions

By eliminating water in its preparation we have succeeded in the synthesis of [Sb(DOTA)]^−^. Interestingly, once made, it is stable towards hydrolysis. This complex is the first metalloid–DOTA complex to be described in the literature and its crystal structure reveals significant differences in comparison to the square antiprismatic geometries of other 8-coordinated complexes, in particular to the like-sized Sc^3+^ complex. Significant geometric distortions for Sb(iii) complexes compared to most metal complexes with the same ligands are common and due to the active lone pair. This lone pair may be important also for the structure and reactivity of [Sb(DOTA)]^−^. HSAB effects can be expected also to be a contributing factor with antimony most likely to prefer ligands containing softer donors than the hard O and N donors provided by DOTA.

Interestingly our work suggests also that the square antiprismatic 8-coordinating cavity offered by DOTA is relatively inflexible and the larger metal ion Bi^3+^ fits more comfortably. Thus, in addition to the active lone pair, a size mismatch for the smaller Sb^3+^ ion may be a contributing factor for its facile replacement by larger ions and remarkably even ones with a lower positive charge. Together all these structural idiosyncrasies rationalize the ease by which the Sb^3+^ can be substituted by other metal ions in water, and not least the bioavailable divalent metal ions, Mg^2+^, Ca^2+^ and Zn^2+^. This is not the case for the stable, clinically applied, like-sized Sc^3+^ complex of DOTA.^62,72^ By supporting stable 6–9 coordinated metal ions in complexes, DOTA has been prolifically applied as a chelating ligand for the metal ions routinely used in PET, SPECT and MRI imaging. The facile substitution reactivity we have described will render, extraordinarily, DOTA practically useless as a chelator for enabling the application of antimony isotopes as theranostic radiopharmaceuticals.

## Experimental methods

### Synthesis

All chemicals and solvents used were reagent grade and were used without further purification unless otherwise stated. SbCl_3_, Y(NO_3_)_3_·6H_2_O, and Sc(ClO_4_)_3_ (40% wt. in H_2_O) were obtained from Sigma-Aldrich, Bi(NO_3_)_3_·5H_2_O was obtained from Fluka, and H_4_DOTA was obtained from Combi-Blocks. Na[Y(DOTA)(OH_2_)]·4H_2_O was prepared using a literature method.^15^

### [(H_6_DOTA)]Cl_2_·4H_2_O·DMSO

H_4_DOTA (15 mg, 0.04 mmol) was dissolved in a hot solution of dilute hydrochloric acid (0.1 M) : DMSO (1 : 1) (2.5 mL). Clear colourless crystals of [(H_6_DOTA)]Cl_2_·4H_2_O·DMSO, suitable for X-ray diffraction, were obtained upon cooling to room temperature.

### Na[Sb(DOTA)]·4H_2_O

H_4_DOTA (88.6 mg, 0.2 mmol) was dissolved in distilled water (5 mL), sodium hydrogen carbonate (73.7 mg, 0.88 mmol) was added, and the mixture was stirred for 4 hours at room temperature. The solvent was removed and the colourless solid (presumed to be Na_4_[DOTA]) was washed with absolute ethanol and collected by filtration. The solid was resuspended in absolute ethanol (3 mL) and a solution of SbCl_3_ (50 mg, 0.2 mmol) dissolved in absolute ethanol (3 mL) was added dropwise. Upon the complete addition of the SbCl_3_ the reaction mixture became clear and colourless, after approximately 10–15 minutes a small amount of the product as colourless precipitate formed. The reaction mixture was heated to approximately 50 °C and stirred for 2 hours before allowing to cool. The colourless solid was collected by filtration. Yield 84 mg, 0.13 mmol, 62%. ESI-MS (pos. mode, MeCN) *m*/*z* 545.0421, (545.06, [HNaSb(DOTA)]^+^, 20%), 567.0266 (567.04 [Na_2_Sb(DOTA)]^+^, 100%); ESI-MS (neg. mode, MeCN) *m*/*z* 521.06 (521.0736 [Sb(DOTA)]^−^, 100%). Anal. Calcd for C_16_H_24_N_4_NaO_8_Sb: C, 35.25; H, 4.44; N, 10.28. Found: C, 35.31; H, 4.83; N, 9.77. Crystals of the tetrahydrate, Na[Sb(DOTA)]·4H_2_O suitable for single crystal X-ray diffraction were obtained by slow diffusion of ethanol into a concentrated solution of the product dissolved in water.

### Na[Bi(DOTA)]·4H_2_O

H_4_DOTA (83.4 mg, 0.206 mmol) and NaOH (33 mg, 0.825 mmol) were dissolved in distilled water (5 mL) and stirred at room temperature for 10 minutes. Bi(NO_3_)_3_·5H_2_O (100 mg, 0.206 mmol) was added in one portion and immediately sonicated until no solids were visible. The resulting mixture as stirred at 80 °C for 2 hours before allowing to cool to room temperature. The volume of the solvent was reduced by half before ethanol (30 mL) was added and a colourless precipitate collected by filtration. Yield 105 mg, 0.15 mmol, 72%. ESI-MS (pos. mode, MeCN) *m*/*z* 655.0968 (655.12, [Na_2_Bi(DOTA)]^+^, 100%);§§It is usual to observe ion charging in positive ion ESI mass spectra by adventitious sodium ions. ESI-MS (neg. Mode, MeCN) *m*/*z* 609.1442 (609.14, [Bi(DOTA)]^−^, 100%). Anal. Calcd for [C_16_H_24_BiN_4_NaO_8_]·H_2_O: C, 29.55; H, 4.03; N, 8.61. Found: C, 30.13; H, 4.28; N, 8.31.

The crystals of Na[Bi(DOTA)]·4H_2_O used for single crystal X-ray diffraction was obtained by the slow diffusion of ethanol over one week into a solution of 5 mg of the solid obtained above redissolved in water (1 mL).

### [H_3_O][Bi(DOTA)]·H_2_O

H_4_DOTA (83.4 mg, 0.206 mmol) was dissolved with stirring in distilled water (3 mL) at room temperature. Bi(NO_3_)_3_·5H_2_O (100 mg, 0.206 mmol) was added in one portion and immediately sonicated until no solids were visible. The resulting mixture was stirred for 2 hours before allowing it to stand at room temperature. After 12 hours the product as colourless crystals were collected. Yield 77 mg, 0.12 mmol, 59%. ESI-MS (pos. mode, MeCN) *m*/*z* 655.0949 (655.12, [Na_2_Bi(DOTA)]^+^, 100%); ESI-MS (neg. mode, MeCN) *m*/*z* 609.1250 (609.14, [Bi(DOTA)]^−^, 100%). Anal. Calcd for C_16_H_27_N_4_BiO_9_: C, 30.58; H, 4.33; N, 8.92. Found: C, 30.14; H, 4.89; N, 8.62.

### Instrumentation

Mass spectra were recorded with Electrospray Ionisation (ESI) on a Bruker micrOTOF-Q II spectrometer (nanospray, capillary temperature = 180 °C, spray voltage = 3.7 kV). NMR spectra were recorded on a Bruker AVANCE III 400 FT spectrophotometer at ambient temperature. X-Ray powder diffraction data were collected from solid samples on a Rigaku MiniFlex 600 diffractometer in the range (2 theta) 5–90° with a step size of 0.02°.

### Sample preparation for mass spectrometry studies

1 mM stock solutions of Na[Sb(DOTA)], Na[Bi(DOTA)], and Na[Y(DOTA)], MgCl_2_·6H_2_O, Ca(NO_3_)_2_·4H_2_O, Y(NO_3_)_3_·6H_2_O, Bi(NO_3_)_3_·5H_2_O, ZnCl_2_, Sb_2_(SO_4_)_3_, and H_4_DOTA were prepared using Milli Q water.

Samples for stability studies were prepared by adding 0.5, 1.0, or 2.0 molar equivalents of MgCl_2_·6H_2_O, Ca(NO_3_)_2_·4H_2_O, ZnCl_2_, Y(NO_3_)_3_·6H_2_O, Bi(NO_3_)_3_·5H_2_O, or Sb_2_(SO_4_)_3_ to aliquots of Na[Sb(DOTA)]·4H_2_O, Na[Bi(DOTA)]·4H_2_O, and Na[Y(DOTA)]·4H_2_O. With the exception of Bi(NO_3_)_3_·5H_2_O, the addition of these aqueous solutions of metal salts did not alter the pH which was close to neutral. The addition of Bi(NO_3_)_3_·5H_2_O to the aqueous solution of [Sb(DOTA)]^−^ lowered the pH to 5–6. The samples were incubated for 2–4 hours at 25 °C before further dilution with acetonitrile (MeCN : H_2_O; 9 : 1) immediately prior to analysis by mass spectrometry. Samples for competition binding study were prepared by adding 0.15 molar equivalents each of MgCl_2_·6H_2_O, Ca(NO_3_)_2_·4H_2_O, ZnCl_2_, Y(NO_3_)_3_·6H_2_O, and Bi(NO_3_)_3_·5H_2_O, and 0.075 molar equivalents of Sb_2_(SO_4_)_3_, to a solution of H_4_DOTA.

The samples were incubated at 25 °C for 2 hours before further dilution with acetonitrile (MeCN : H_2_O; 9 : 1) immediately prior to analysis by mass spectrometry.

### Crystallography

Crystals used for SCXRD were taken directly from the mother liquor and coated in Fomblin®Y or Paratone oil to allow the crystal to adhere to the mounting loop. All the data sets were collected at 100(1)K on a Synergy, Dualflex, AtlasS2 diffractometer with the *CrysAlis PRO* suite,^84^ using CuKα radiation for [H_6_DOTA]Cl_2_·4H_2_O·DMSO and MoKα radiation for the structures containing heavy atoms. The structures were solved by dual space methods (SHELXT^85^) and refined on *F*^2^ using all the reflections (SHELXL-2018/3 (ref. 86)), using shelXle^87^ and Olex2.^88^ Except where noted in the ESI† for each structure, all the non-hydrogen atoms were refined using anisotropic atomic displacement parameters; hydrogen atoms bonded to carbon were inserted at calculated positions using a riding model, and those bound to O or N were located from difference maps and their coordinates refined. Crystal parameters, data collection and structure refinement details are summarized in Table 2.

**Table 2:** Crystallographic data

	[H_6_DOTA]Cl_2_·4H_2_O·DMSO	Na[Sb(DOTA)]·4H_2_O	Na[Bi(DOTA)]·4H_2_O	[H_3_O][Bi(DOTA)]·H_2_O
Empirical formula	C_18_H_44_N_4_O_13_SCl_2_	C_32_H_64_N_8_O_24_Na_2_Sb_2_	C_32_H_64_N_8_O_24_Na_2_Bi_2_	C_16_H_29_N_4_O_10_Bi
Formula weight	627.53	1234.39	1408.85	646.41
Temperature/K	100.00(10)	100.01(10)	100.01(10)	100.00(10)
Crystal system	Monoclinic	Monoclinic	Monoclinic	Tetragonal
Space group	*P*2_1_/*n*	*P*2/*c*	*P*2/*c*	*P*4/*n*
*A*/Å	11.1185(2)	17.0714(2)	17.1003(2)	12.13250(10)
*B*/Å	21.3172(4)	6.51830(10)	6.55970(10)	12.13250(10)
*C*/Å	12.1038(2)	19.8040(2)	19.8702(3)	6.56530(10)
*α*/°	90	90	90	90
*β*/°	90.5130(10)	101.6130(10)	101.6200(10)	90
*γ*/°	90	90	90	90
Volume/Å^3^	2868.67(9)	2158.61(5)	2183.22(5)	966.40(2)
*Z*	4	2	2	2
*ρ*_calc_/g cm^−3^	1.453	1.899	2.143	2.221
*μ*/mm^−1^	3.310	1.373	8.169	9.190
*F*(000)	1336.0	1256.0	1384.0	632.0
Wavelength/Å	1.54184	0.71073	0.71073	0.71073
2*θ* range/°	8.296 to 149.338	5.758 to 61.134	5.248 to 61.188	6.206 to 61.158
Reflections collected	26 196^a^	91 865	93 107	41 527
Independent refl. [*R*_int_]	9137 [0.0632^a^]	6359 [0.0328]	6353 [0.0354]	1468 [0.0360]
Data/restraints/param	9137/15/393	6359/14/332	6353/14/333	1468/66/94
Goodness-of-fit on *F*^2^	0.997	1.116	1.065	1.116
*R*_1_, w*R*_2_ [*I* > 2*σ*(*I*)]	0.0447, 0.1258	0.0192, 0.0496	0.0139, 0.0272	0.0109, 0.0233
*R*_1_, w*R*_2_ [all data]	0.0590, 0.1310	0.0222, 0.0510	0.0169, 0.0280	0.0116, 0.0235
Max Δ peak/hole/e Å^−3^	1.08/−0.91	0.57/−0.84	0.59/−0.60	0.51/−0.57

a For HKLF4 data.

## Conflicts of interest

There are no conflicts to declare.

## Supplementary Material

RA-012-D2RA00642A-s001

RA-012-D2RA00642A-s002
