# Supplementary material for: Facile transmetallation of [SbIII(DOTA)]− renders it unsuitable for medical applications
Source: RSC Adv. 2022 Feb 16;12(10):5772–81. doi: 10.1039/d2ra00642a (PMC8981601; doi:10.1039/d2ra00642a)
Supplement: RA-012-D2RA00642A-s001 [file RA-012-D2RA00642A-s001.pdf]

Electronic Supplementary Material (ESI) for RSC Advances.
This journal is © The Royal Society of Chemistry 2022

Supplementary Information

Facile Transmetallation of $[Sb^{III}(DOTA)]^-$ renders it unsuitable for medical applications

Catherine Chen, Charlotte Sommer, Helge Thisgaard, Vickie McKee, and Christine J. McKenzie

Index

1. Mass spectrometry 3

Figure S1. Expanded ESI mass spectrum of $Na[Sb(DOTA)]\cdot4H_2O$ 3

Figure S2. ESI mass spectra of $Na[Bi(DOTA)]\cdot4H_2O$ 3

Figure S3. ESI mass spectra of Na[Y(DOTA)] 4

Figure S4 ESI mass spectra of $[H_4DOTA]$ incubated with M^{3+} (Bi, Sb, and Y) and M^{2+} (Ca, Mg, Zn) ions 4

Figure S5. ESI Spectra of competition study between $Na[Sb(DOTA)\cdot4H_2O$ and M^{3+} (M=Bi and Y) metal ions. 5

Figure S6. ESI Spectra of competition study between $Na[Sb(DOTA)]\cdot4H_2O$ and Sc^{3+} ions. 6

Figure S7. ESI Spectra of competition study between $Na[Sb(DOTA)]\cdot4H_2O$ and Zn^{2+} ions. 7

Figure S8. ESI Spectra of competition study between $Na[Sb(DOTA)]\cdot4H_2O$ and Ca^{2+} ions. 8

Figure S9. ESI Spectra of competition study between $Na[Sb(DOTA)]\cdot4H_2O$ and Mg^{2+} ions. 9

Figure 10. ESI Spectra of competition study between $Na[Y(DOTA)]\cdot4H_2O$ and M^{3+} (M= Bi and Sb) ions. 10

Figure 11. ESI Spectra of competition study between $Na[Y(DOTA)]\cdot4H_2O$ and M^{2+} (M= Ca, Mg and Zn) ions. 11

Figure 12. ESI Spectra of competition study between $Na[Bi(DOTA)]\cdot4H_2O$ and M^{3+} (M= Sb and Y) ions. 12

Figure 13. ESI Spectra of competition study between $Na[Bi(DOTA)]\cdot4H_2O$ and M^{2+} (M= Ca, Mg and Zn) ions. 13

2. PXRD 14

Figure S14. PXRD of (a) $[H_3O][Bi(DOTA)]\cdot H_2O$ and (b) $Na[Bi(DOTA)]\cdot4H_2O$ 14

Figure S15. PXRD of $Na[Sb(DOTA)]\cdot4H_2O$ 14

3. Crystallography 15

$[H_6DOTA]Cl_2\cdot4H_2O\cdot DMSO$: 15

Figure S16. H-bonding network in $[H_6DOTA]Cl_2\cdot4H_2O\cdot DMSO$. 15

Figure S17. Packing diagram for $[H_6DOTA]Cl_2\cdot4H_2O\cdot DMSO$ viewed down the c axis 15

Figure S18. Overlays of the DOTA 16

Table S1. Hydrogen-bond geometry (Å, °) for $[H_6DOTA]Cl_2\cdot4H_2O\cdot DMSO$ 16

Na[Sb(DOTA)]·4H2O and Na[Bi(DOTA)]·4H2O 18

Table S2. Lattice type analyses for $Na[Sb(DOTA)]\cdot4H_2O$ and $Na[Bi(DOTA)]\cdot4H_2O$. 18

Figure S19. Asymmetric unit of $Na[Sb(DOTA)]\cdot4H_2O$, ... 19

Figure S20. Packing diagram of Na[Sb(DOTA)]·4H_2O viewed down the b axis, showing OH---O H bonds 19
Figure S21. Packing diagram of Na[Sb(DOTA)]·4H_2O viewed down the b axis, showing CH---O H bonds 20
Figure S22. Asymmetric unit of Na[Bi(DOTA)]·4H_2O 20
Figure S23. $[Bi(DOTA)]^-$ (top) and $[(Na(H_2O)_4)_2Bi(DOTA)]^+$(bottom) 21
Table S3. Hydrogen-bond geometry (Å, °) for Na[Sb(DOTA)]·4H_2O 22
Table S4. Hydrogen-bond geometry (Å, °) for Na[Bi(DOTA)]·4H_2O 23
$[H_3O][Bi(DOTA)]\cdot H_2O$ 24
Figure S24. $[H_3O][Bi(DOTA)]\cdot H_2O$ showing disorder in the carboxylate groups and the H_3O+/H_2O 25
Figure S25. Packing diagram for $[H_3O][Bi(DOTA)]\cdot H_2O$ viewed down the c axis. Hydrogen bonds 25
Table S5. Hydrogen-bond geometry (Å, °) for $[H_3O][Bi(DOTA)]\cdot H_2O$ 26
Figure S26. Overlay of the structures of $[Sc(DOTA)]^-$ (pale blue from LUQCIJ) and $[Sb(DOTA)]^-$(purple) 26
Table S6. Expanded version of table 1 with selected bond lengths (Å) for Na[Sb(DOTA)]·4H_2O and Na[Bi(DOTA)]·4H_2O and structures from literature containing $[Sc(DOTA)]^-$.[a] 27

1. Mass spectrometry

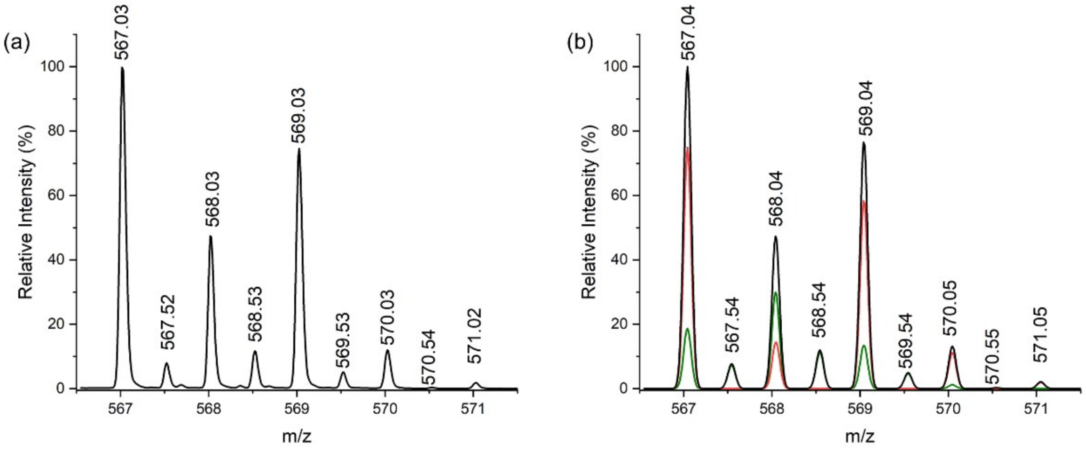

Figure S1. (a) Expanded section of ESI mass spectra for Na[Sb(DOTA)]·4H_2O recorded in positive mode (Figure 2(a)) showing the isotope pattern for peak m/z 567.03 and **(b)** the calculated isotope patterns for $[Na_2Sb(DOTA)]^+$ (red), $\{[Na_2Sb(DOTA)]_2\}^{2+}$ (blue) and the corresponding combined spectra of $[Na_2Sb(DOTA)]^+$ and $\{[Na_2Sb(DOTA)]_2\}^{2+}$ (black).

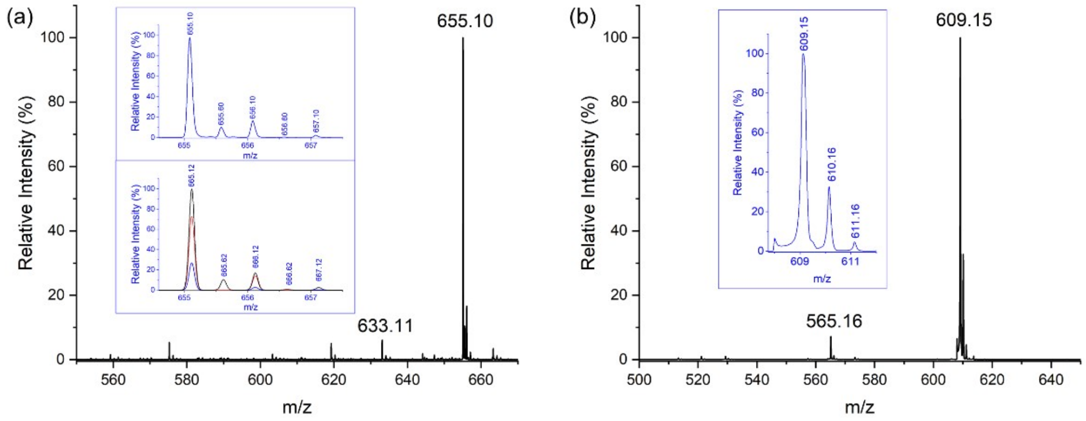

Figure S2. ESI mass spectra of Na[Bi(DOTA)·4H_2O. in water: acetonitrile (1:50) recorded in **(a)** positive mode show peaks corresponding to $[NaHBi(DOTA)]^+$ (m/z 633.11) and $[Na_2Bi(DOTA)]^+$ overlayed with a smaller fraction of $\{[Na_2Bi(DOTA)]_2\}^{2+}$ (m/z 655.10) (expanded in inset: found spectra (top) compared to the fitted spectra (bottom)), and **(b)** negative mode showing peaks corresponding to $\{[Bi(DOTA)]\text{-}CO_2\}^-$ (m/z 565.16) and $[Bi(DOTA)]^-$ (m/z 609.15) (expanded in inset).

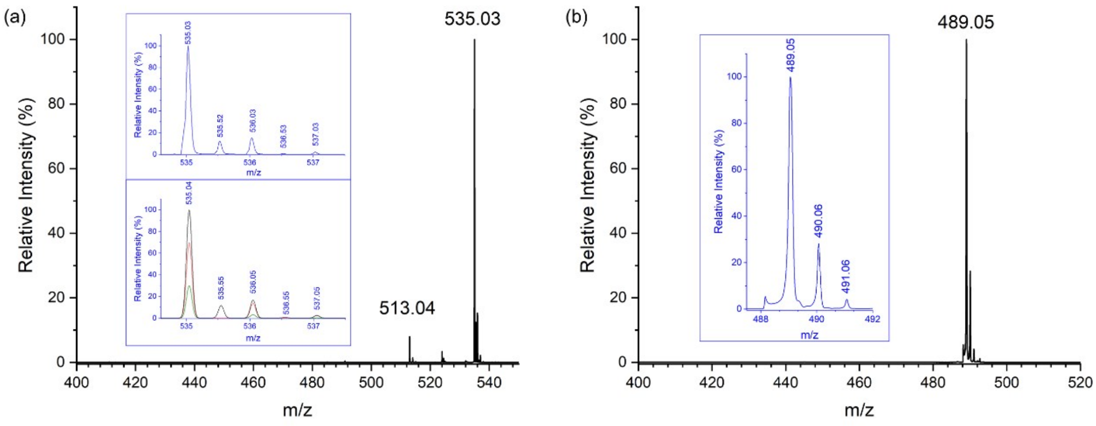

Figure S3. ESI mass spectra of Na[Y(DOTA)·4H_2O. in water: acetonitrile (1:50) recorded in **(a)** positive mode show peaks corresponding to $[NaHY(DOTA)]^+$ (m/z 513.04) and $[Na_2Bi(DOTA)]^+$overlayed with a smaller fraction of $\{[Na_2Y(DOTA)]_2\}^{2+}$ (m/z 535.03) (expanded in inset: found spectra (top) compared to the fitted spectra (bottom)), and **(b)** negative mode showing one peak corresponding to $[Y(DOTA)]^-$ (m/z 489.05) (expanded in inset).

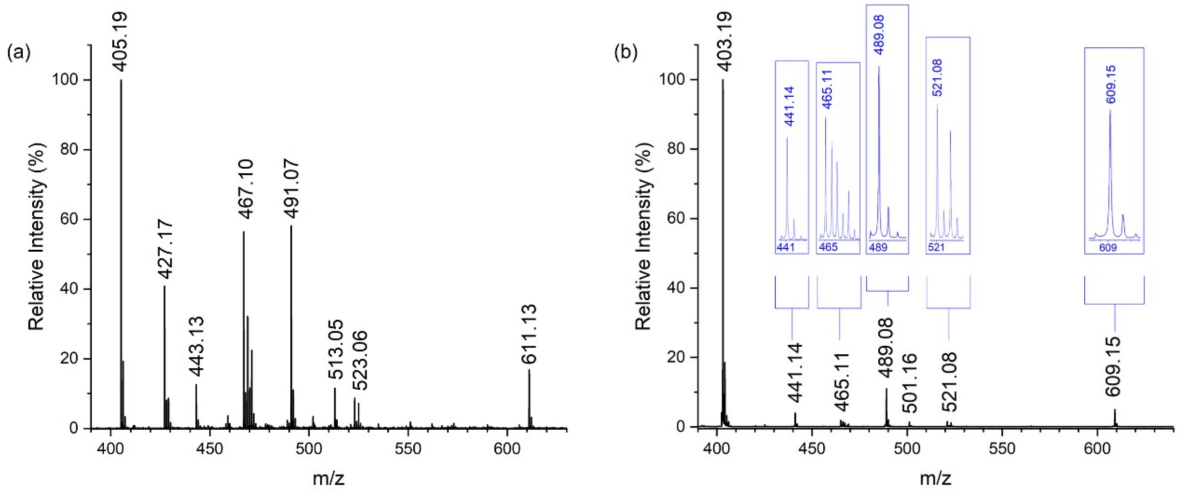

Figure S4. ESI mass spectra of samples containing acetonitrile: water (9:1). Samples were diluted from aqueous solutions of $[H_4DOTA]$ (1mM) incubated with $Ca(NO_3)_2$·4H_2O (0.15 mM), $MgCl_2$·6H_2O (0.15 mM), $ZnCl_2$ (0.15 mM), $Y(NO_3)_3$·6H_2O (0.15 mM), $Bi(NO_3)_3$·5H_2O (0.15 mM), and $Sb_2(SO_4)_3$ (0.075 mM) for 2 hours at 298K and recorded in; **(a)** Positive mode showing peaks corresponding to $[H_5DOTA]^+$ (m/z 405.19), $[H_3Mg(DOTA)]^+$ (m/z 427.17), $[H_3Ca(DOTA)]^+$ (m/z 443.13), $[H_3Zn(DOTA)]^+$ (m/z 461.10), $[H_2Y(DOTA)]^+$ (m/z 491.07), $[NaHY(DOTA)]^+$ (m/z 513.05), $[H_2Sb(DOTA)]^+$ (m/z 523.06), and $[H_2Bi(DOTA)]^+$ (m/z 611.13). **(b)** Negative mode showing peaks corresponding to $[H_3DOTA]^-$(m/z 403.19), $[HCa(DOTA)]^-$(m/z 441.14), $[HZn(DOTA]^-$(m/z 465.11), $[Y(DOTA)]^-$(m/z 489.08), $[Sb(DOTA)]^-$ (m/z 521.08), and $[Bi(DOTA)]^-$ (m/z 609.15).

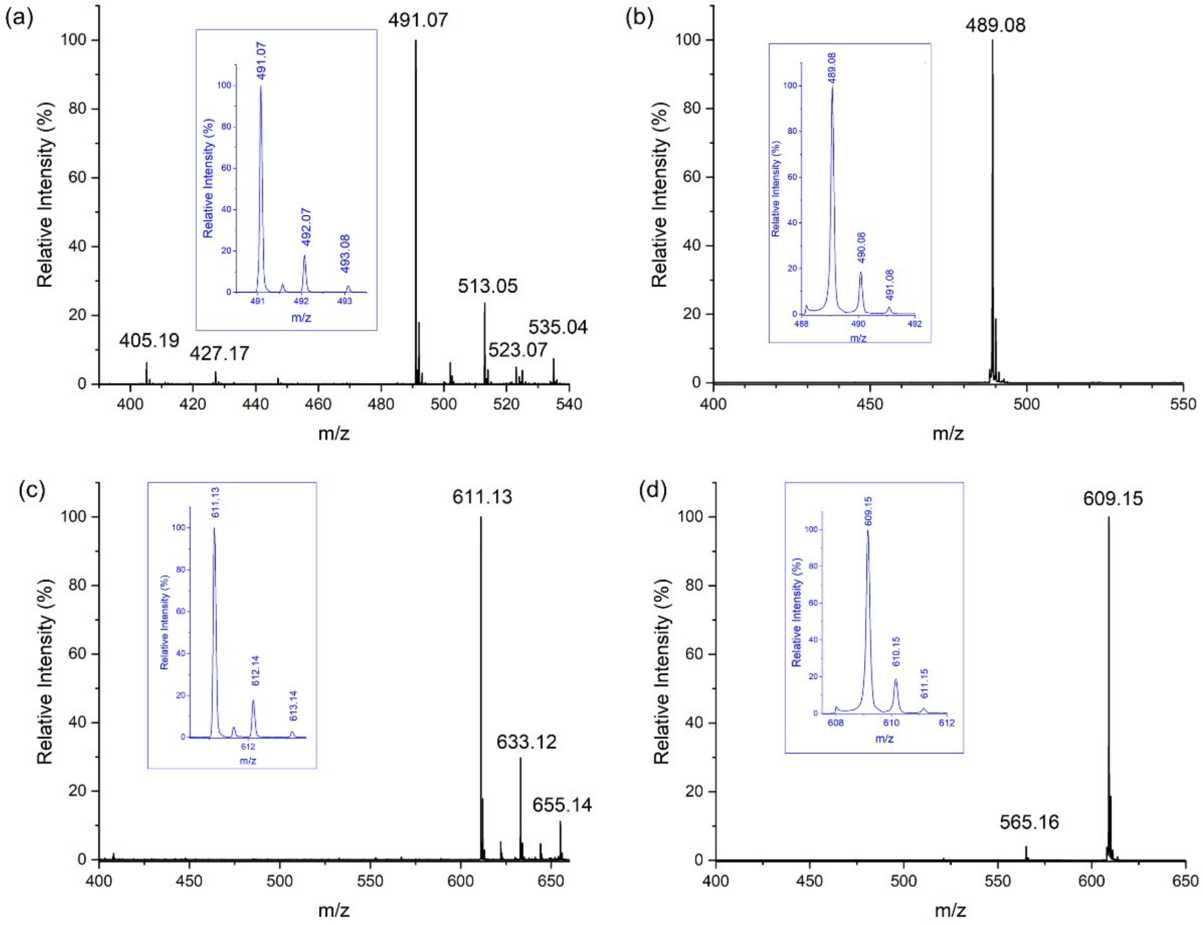

Figure S5. ESI mass spectra of samples containing acetonitrile: water (9:1). Samples were diluted from an aqueous solution containing Na[Sb(DOTA)·4H_2O (0.33 mM) incubated with M^{3+} (M= Y, Bi) (0.67 mM) at 298 K for at least 2 hours. The replacement of Sb^{3+} by Y^{3+} was recorded in; (a) positive ESI mode showing peaks corresponding to $[H_2Y(DOTA)]^+$ (m/z 491.07)(expanded in inset), $[NaHY(DOTA)]^+$ (m/z 513.05), and $[Na_2Y(DOTA)]^+$ (m/z 535.04) and (b) negative ESI mode showing peaks corresponding to $[Y(DOTA)]^-$ (m/z 489.08)(expandd in inset). The replacement of Sb^{3+} with Bi^{3+} was recorded in; (c) positive ESI mode showing peaks corresponding to $[H_2Bi(DOTA)]^+$ (m/z 611.13)(expanded in inset), $[NaHBi(DOTA)]^+$ (m/z 633.12), and $[Na_2Bi(DOTA)]^+$ (m/z 655.14), and (d) negative ESI mode showing peaks corresponding to $[Bi(DOTA)]^-$ (m/z 609.15) and $\{[Bi(DOTA)]-CO_2\}^-$ (m/z 565.15)(expanded in inset).

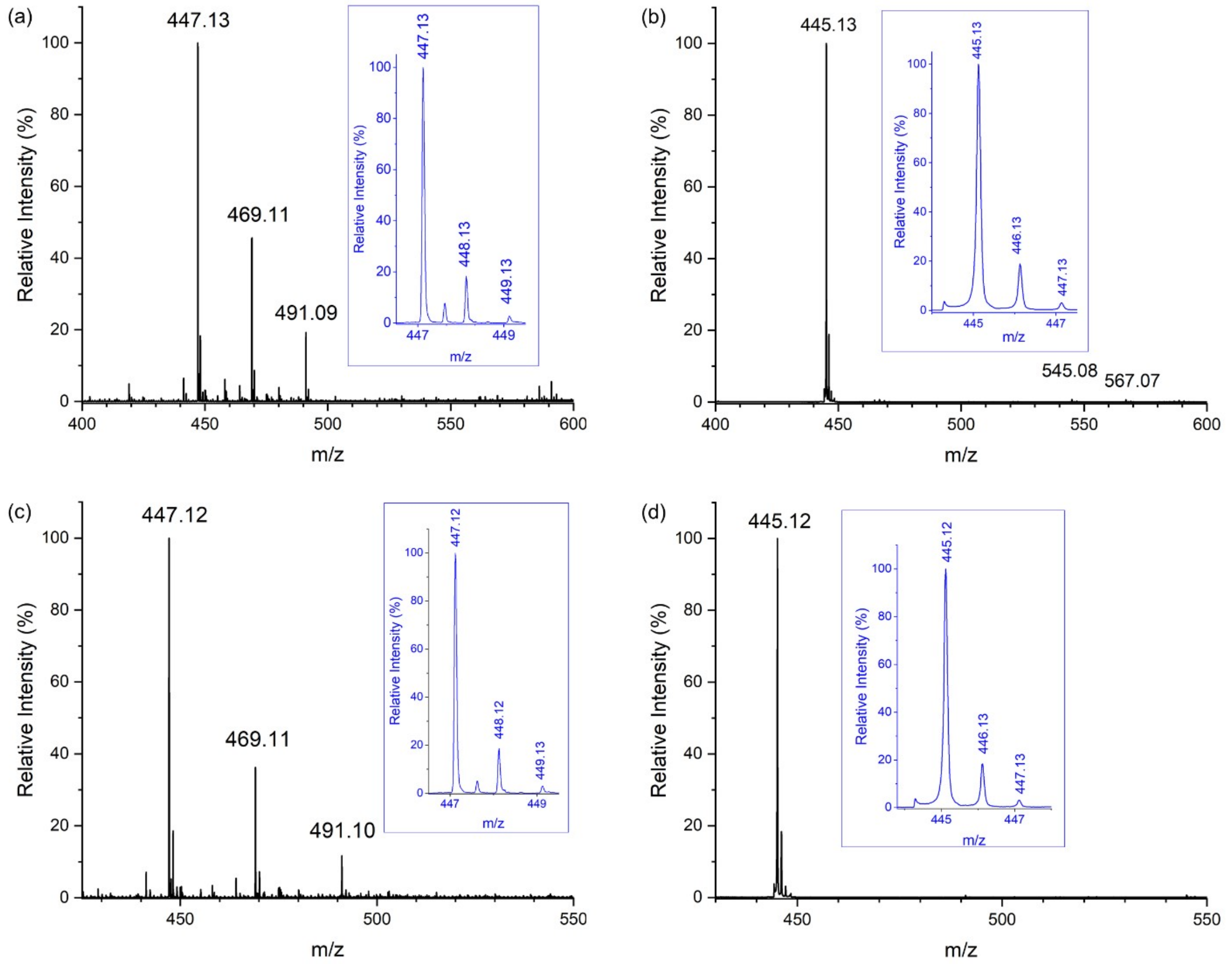

Figure S6. ESI mass spectra of samples containing acetonitrile: water (9:1). Samples were diluted from an aqueous solution containing Na[Sb(DOTA)·4H_2O (0.5 mM) and $Sc(ClO_4)_3$ (0.5 mM) incubated at 298 K showing the replacement of Sb^{3+} with Sc^{3+} recorded in; **(a)** positive mode with peaks corresponding to $[H_2Sc(DOTA)]^+$ (m/z 447.13) (expanded in inset), $[NaHSc(DOTA)]^+$ (m/z 469.11), and $[Na_2Sc(DOTA)]^+$ (m/z 491.09) and **(b)** negative mode with peaks corresponding to $[Sc(DOTA)]^-$ (m/z 445.13) (expanded in inset), $[NaHSb^{I}(DOTA)]^-$ (m/z 545.08), and $[Na_2Sb^{I}(DOTA)]^-$ (m/z 567.07). Samples were diluted from an aqueous solution containing Na[Sb(DOTA)·4H_2O (0.33 mM) and $Sc(ClO_4)_3$ (0.67 mM) incubated at 298 K showing the replacement of Sb^{3+} with Sc^{3+} recorded in; **(c)** positive mode with peaks corresponding to $[H_2Sc(DOTA)]^+$ (m/z 447.13) (expanded in inset), $[NaHSc(DOTA)]^+$ (m/z 469.11), and $[Na_2Sc(DOTA)]^+$ (m/z 491.09) and **(d)** negative mode with peaks corresponding to $[Sc(DOTA)]^-$ (m/z 445.13) (expanded in inset).

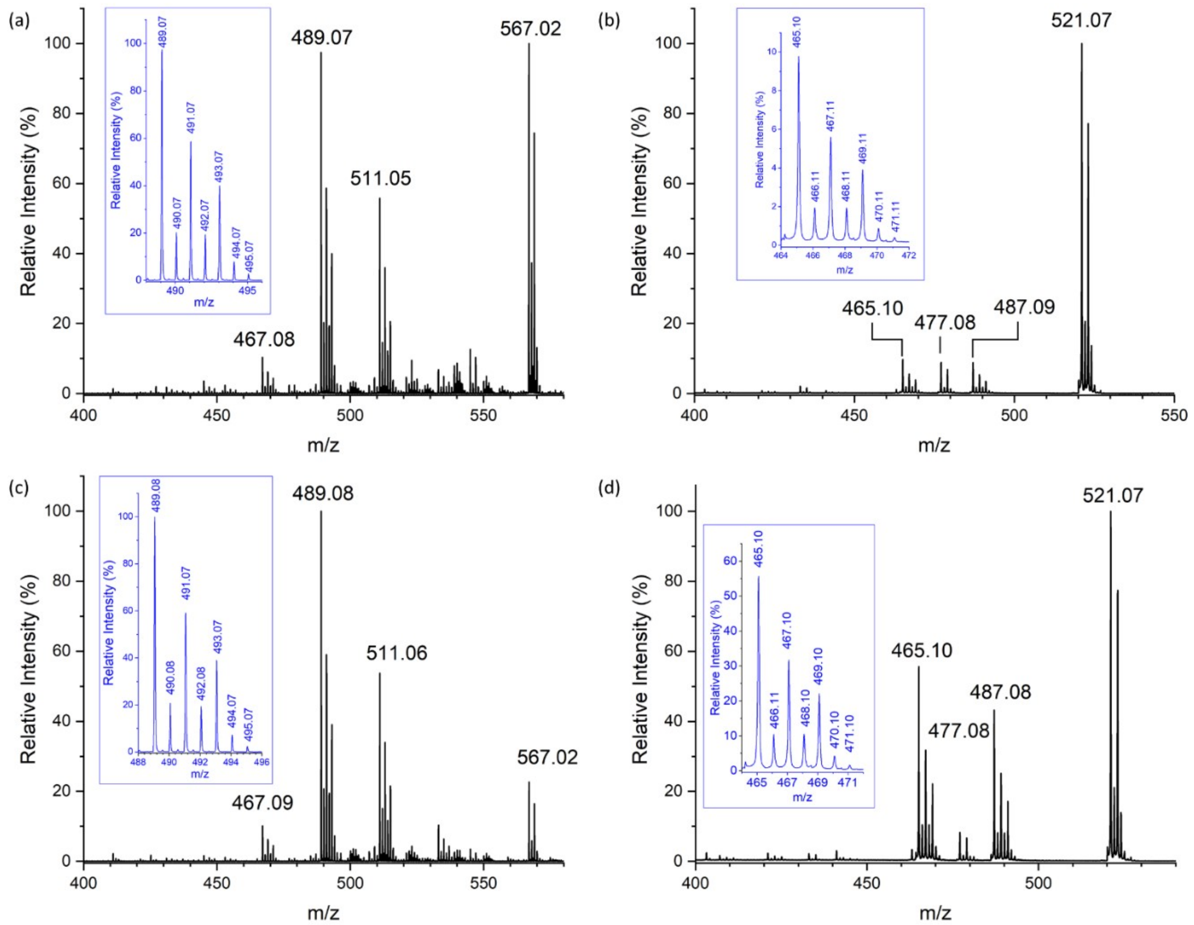

Figure S7. ESI mass spectra of samples containing acetonitrile: water (9:1). Samples were diluted from aqueous solutions of Na[Sb(DOTA)·4H_2O (0.5 mM) incubated with one molar equivalents of $ZnCl_2$ (0.5 mM) for 2 hours at 298K and recorded in; **(a)** Positive mode showing peaks corresponding to $[H_3Zn(DOTA)]^+$ (m/z 467.08), $[H_2NaZn(DOTA)]^+$ (m/z 489.07) (expanded in inset), $[HNa_2Zn(DOTA)]^+$ (m/z 511.05), $[Na_2Sb(DOTA)]^+$ (m/z 567.02) **(b)** Negative mode showing peaks corresponding to $[HZn(DOTA]^-$(m/z 465.10) (expanded in inset), $\{[Sb(DOTA)]-CO_2\}^-$(m/z 477.08), and $[NaZn(DOTA)]^-$ (m/z 487.09) and $[Sb(DOTA)]^-$ (m/z 521.07).

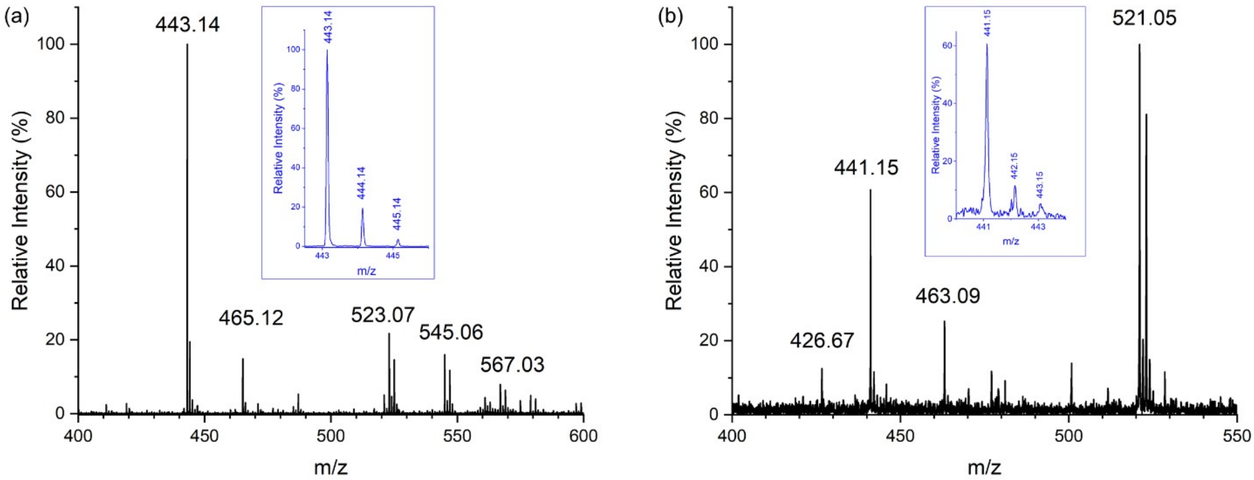

Figure S8. ESI mass spectra recorded in negative mode of samples containing acetonitrile: water (9:1). Samples were diluted from aqueous solutions containing Na[Sb(DOTA)·4H_2O (0.5mM) incubated with $Ca(NO_3)_2$·4H_2O (0.5 mM) at 298K showing the replacement of Sb^{3+} with Ca^{2+} recorded in; (a) Positive mode with peaks corresponding to $[H_3Ca(DOTA)]^+$ (m/z 443.14) (expanded in inset), $[NaH_2Ca(DOTA)]^+$ (m/z 465.12), $[H_2Sb(DOTA)]^+$ (m/z 523.07), $[NaHSb(DOTA)]^+$ (m/z 545.06), and $[Na_2Sb(DOTA)]^+$ (m/z 567.03). (b) Negative mode with peaks corresponding to $[HCa(DOTA)]^-$ (m/z 441.14) (expanded in inset), $[NaCa(DOTA)]^-$ (m/z 463.12), and $[Sb(DOTA)]^-$ (m/z 521.07).

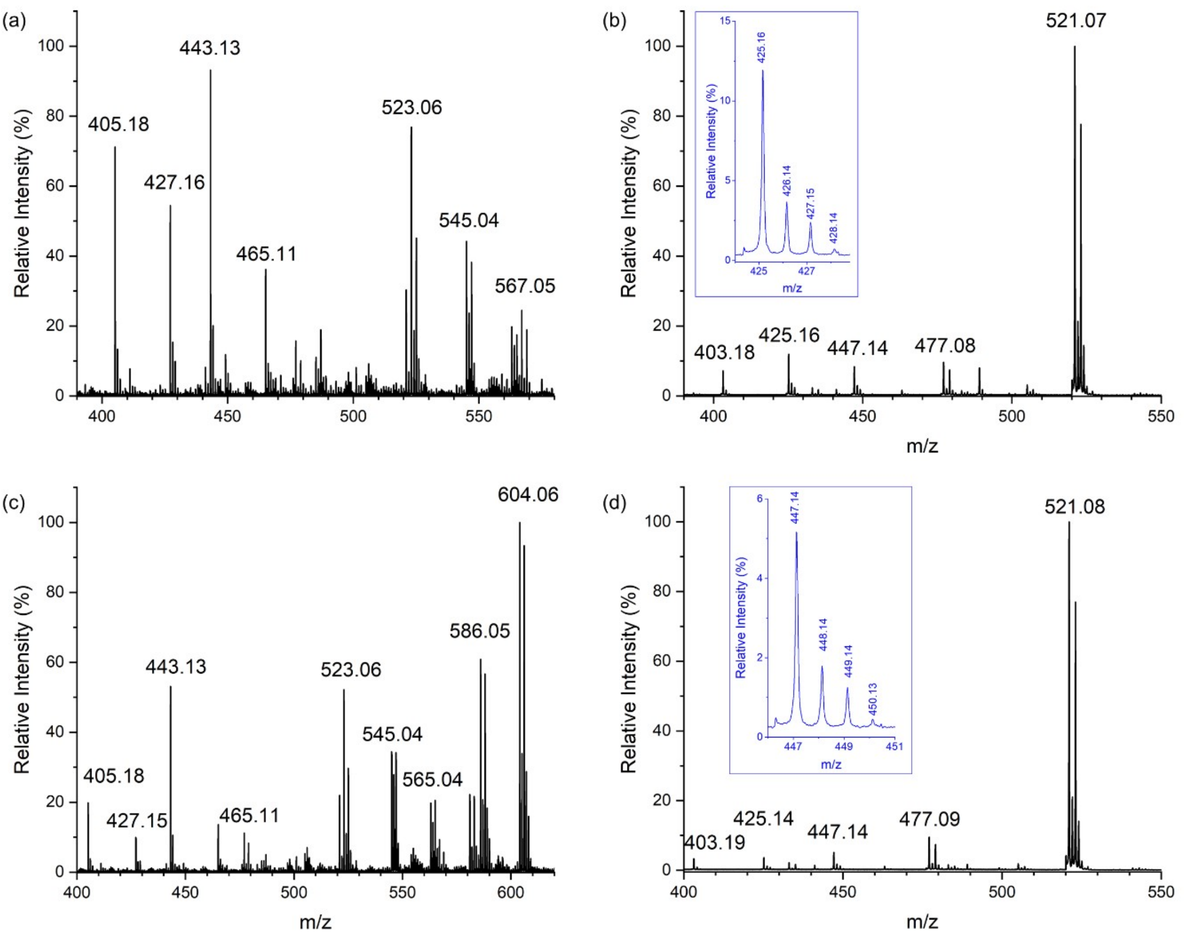

Figure S9. ESI mass spectra of samples containing acetonitrile: water (9:1). Samples were diluted from aqueous solutions of Na[Sb(DOTA)·$4H_2O$ (1 mM) incubated with one molar equivalents of $MgCl_2 \cdot 6H_2O$ for 2 hours at 298K and recorded in; **(a)** Positive mode showing peaks corresponding to $[H_3Mg(DOTA)]^+$ (m/z 427.16), $[H_2Sb(DOTA)]^+$ (m/z 523.06), $[HNaSb(DOTA)]^+$ (m/z 545.04), $[Na_2Sb(DOTA)]^+$ (m/z 567.02) **(b)** Negative mode showing peaks corresponding to $[HMg(DOTA]^-$(m/z 425.16) (expanded in inset), $[NaMg(DOTA]^-$(m/z 447.14), $\{[Sb(DOTA)]\text{-}CO_2\}^-$(m/z 477.08), and $[Sb(DOTA)]^-$ (m/z 521.07). Incubation with two molar equivalents of $MgCl_2.6H_2O$ did not result in an observable increase in substitution of Sb^{3+} by Mg^{2+} in ESI mass spectra in both **(c)** positive and **(d)** negative modes.

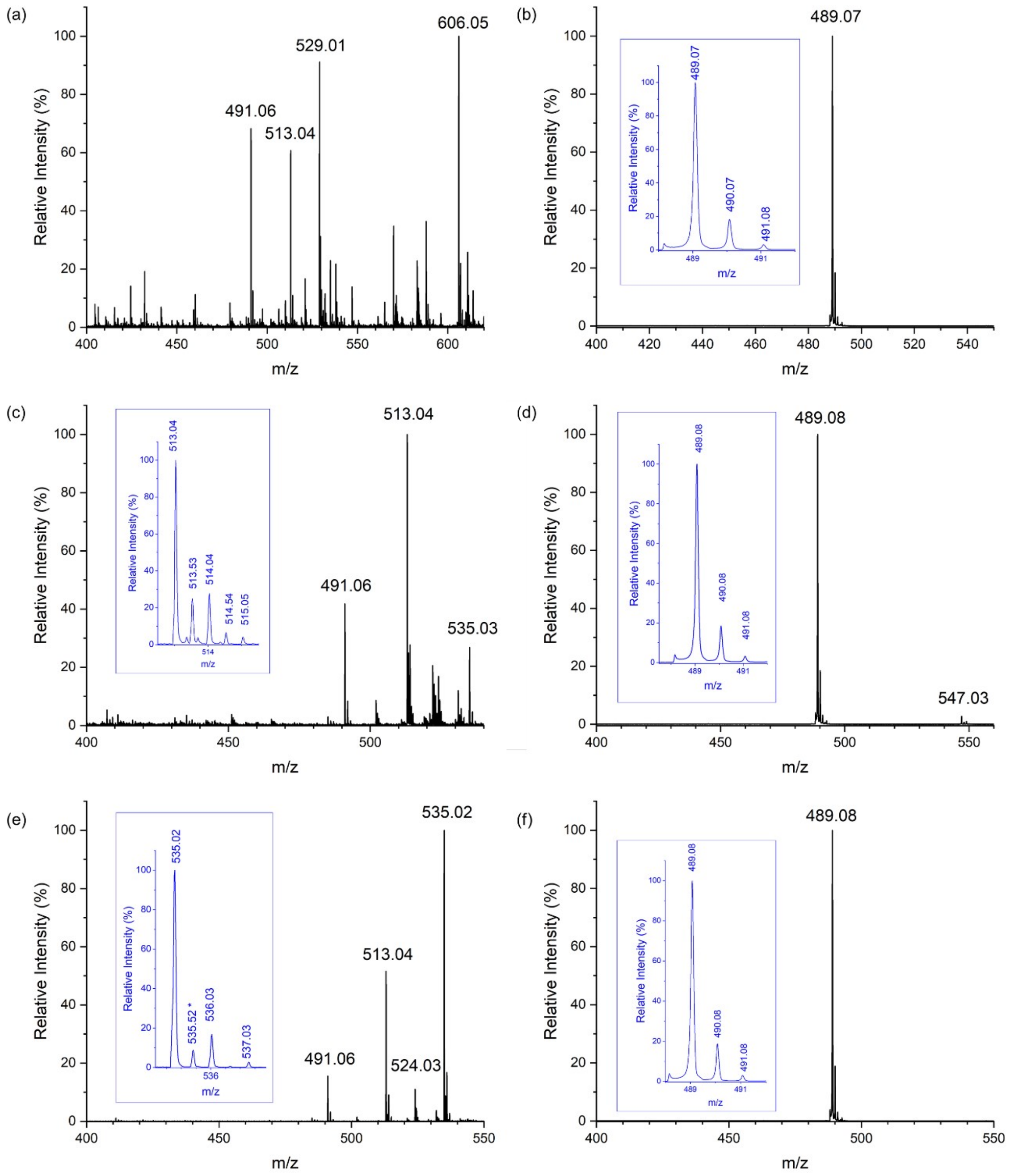

Figure S10. ESI mass spectra recorded in negative mode of samples containing acetonitrile: water (9:1). Samples were diluted from aqueous solutions containing Na[Y(DOTA)·4H_2O (0.33 mM) incubated with 2 molar equivalents of M^{2+}(0.67 mMol) (M=Ca, Mg, Zn) ions. Incubation with $Ca(NO_3)_2$·4H_2O (0.67 mM) recorded in; **(a)** ESI positive mode and **(b)** ESI negative mode did not result in any observable replacement of Y^{3+} with Ca^{2+}, peak corresponding to $[CaY(DOTA)]^+$ (m/z 529.01). Incubation with $MgCl_2$·6H_2O (0.67 mM) recorded in; **(c)** ESI positive and **(d)** ESI negative mode did not result in any observable replacement of Y^{3+} with Mg^{2+}. Incubation with $ZnCl_2$ (0.67 mM) recorded in; **(e)** ESI positive mode and **(f)** ESI negative mode did not result in any observable replacement of Y^{3+} with Zn^{2+}. ESI positive spectra show peaks corresponding to $[H_2Y(DOTA)]^+$ (m/z 491.06) (expanded in inset), $[NaHY(DOTA)]^+$ (m/z 513.04) (expanded in inset), and $[Na_2Y(DOTA)]^+$ (m/z 535.02). ESI negative spectra showing peaks corresponding to $[Y(DOTA)]^-$ (m/z 489.08) (expanded in inset).

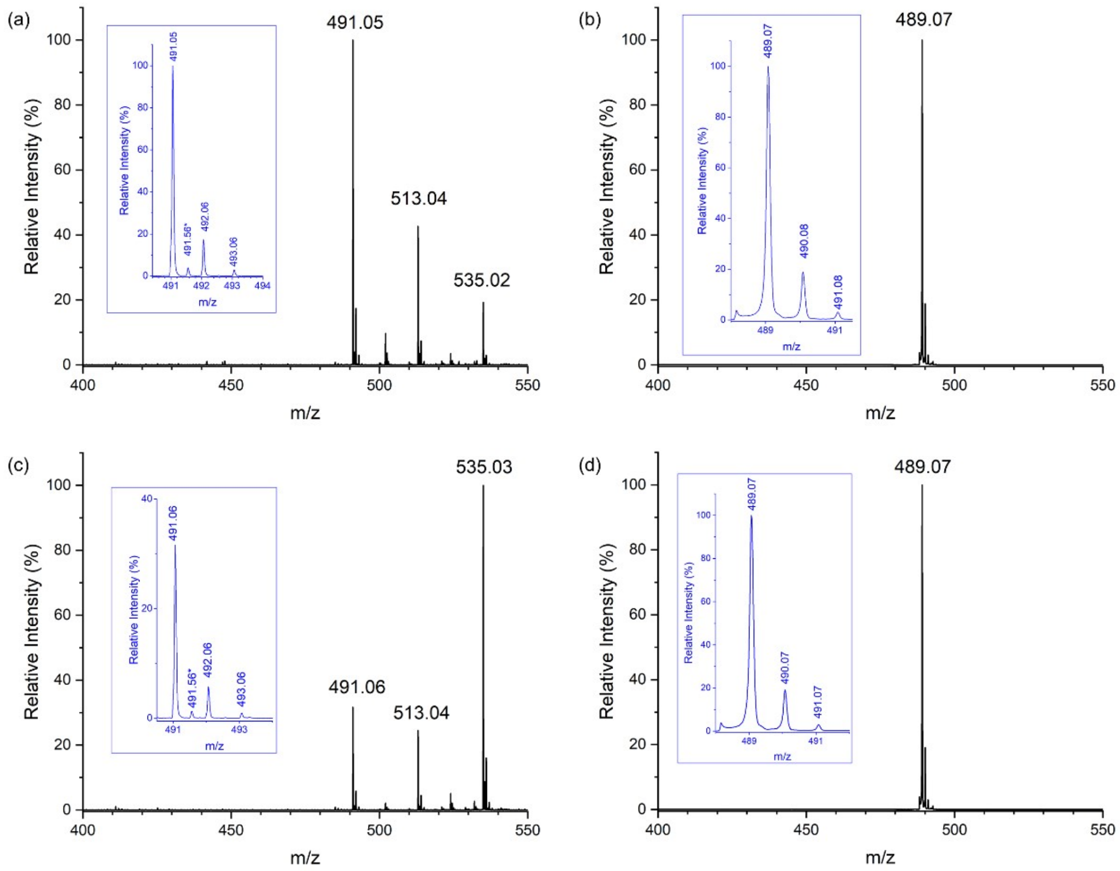

Figure S11. ESI mass spectra recorded in negative mode of samples containing acetonitrile: water (9:1). Samples were diluted from aqueous solutions containing Na[Y(DOTA)·4H_2O (0.33 mM) incubated with 2 molar equivalents of M^{3+}(0.67 mMol) (M=Bi and Sb) for at least 2 hours at 298 K. The ESI mass spectra of the incubation with $Bi(NO_3)_3.5H_2O$ recorded in **(a)** positive and **(b)** negative modes did not result in any substitution of the Y^{3+} in $[Y(DOTA)]^-$. The ESI mass spectra of the incubation with $Sb_2(SO_4)_3$ recorded in **(c)** positive and **(d)** negative modes did not result in any substitution of the Y^{3+} in $[Y(DOTA)]^-$. Only peaks corresponding to $[H_2Y(DOTA)]^+$ (m/z 491.05) (expanded in inset), $[NaHY(DOTA)]^+$ (m/z 513.04), and $[Na_2Y(DOTA)]^+$ (m/z 535.03) were observed in ESI positive mass spectra, and $[Y(DOTA)]^-$ (m/z 489.07) (expanded in inset) were observed in ESI negative spectra.

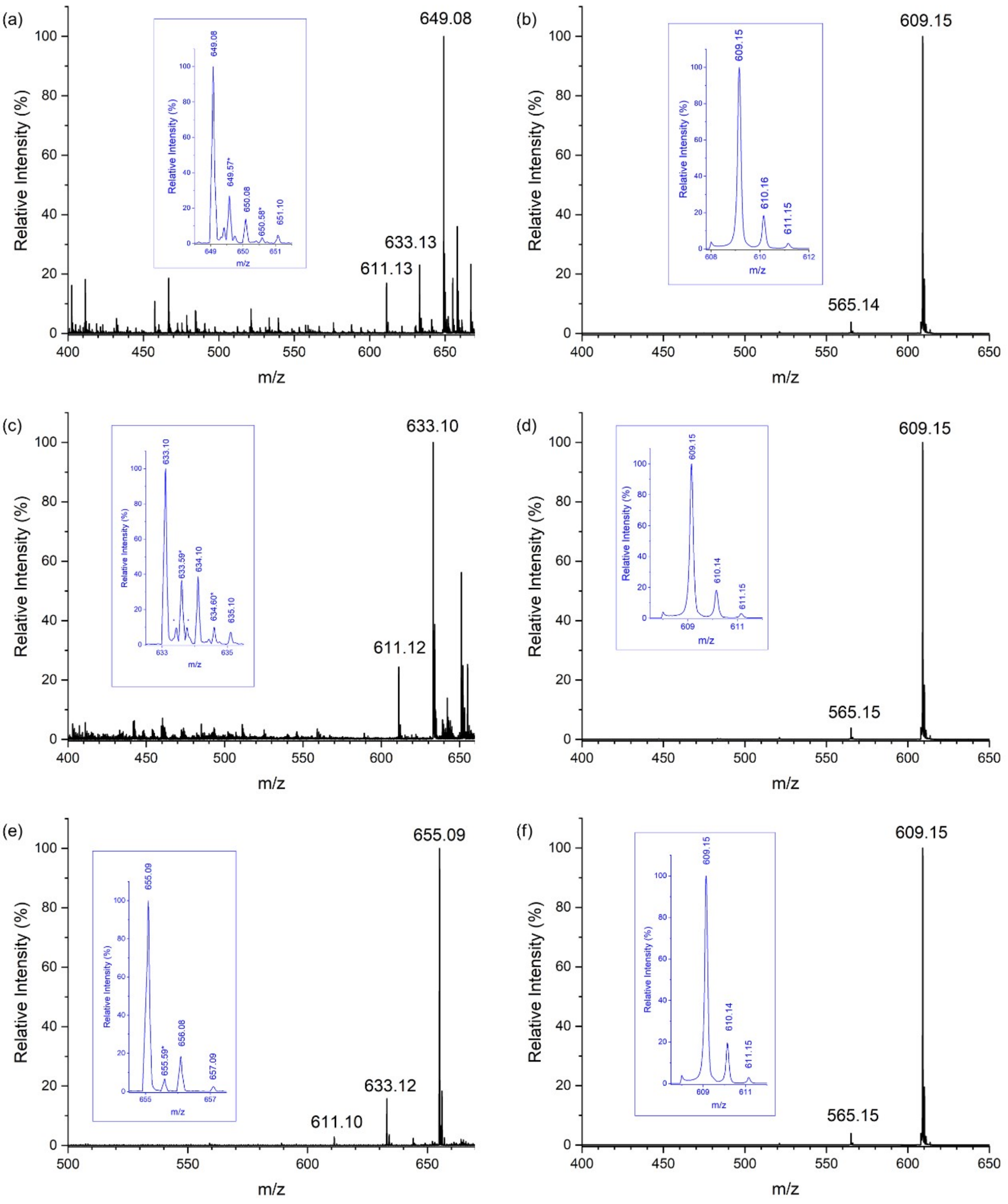

Figure S12. ESI mass spectra recorded in negative mode of samples containing acetonitrile: water (9:1). Samples were diluted from aqueous solutions containing Na[Bi (DOTA)·4H_2O (0.33 mM) incubated with 2 molar equivalents of M^{2+}(M=Ca, Mg, and Zn) (0.67 mMol) for at least 2 hours at 298 K. The incubation of Na[Bi(DOTA)·4H_2O with $Ca(NO_3)_2$·5H_2O, $MgCl_2$·6H_2O, and $ZnCl_2$ did not result in any observable substituton of Bi^{3+} with Ca^{2+}, Mg^{2+} or Zn^{2+} as observed in the ESI mass spectra recorded in positive mode **(a)**, **(c)**, and **(e)** respectively, or in negative mode **(b)**, **(d)** and **(f)** respectively. Peaks observed corresponding to $[H_2Bi(DOTA)]^+$ (m/z 611.10), $[NaHBi(DOTA)]^+$ (m/z 633.10) (expended in inset), $[Na_2Bi(DOTA)]^+$ (m/z 655.09) (expanded in inset), and $[CaBi(DOTA)]^+$ (m/z 649.08) (expanded in inset) were observed in ESI positive mass spectra and peaks corresponding to [Bi(DOTA)] (m/z 609.15) and $\{[Bi(DOTA)]-CO_2\}^-$ (m/z 565.15) were observed in ESI negative mass spectra.

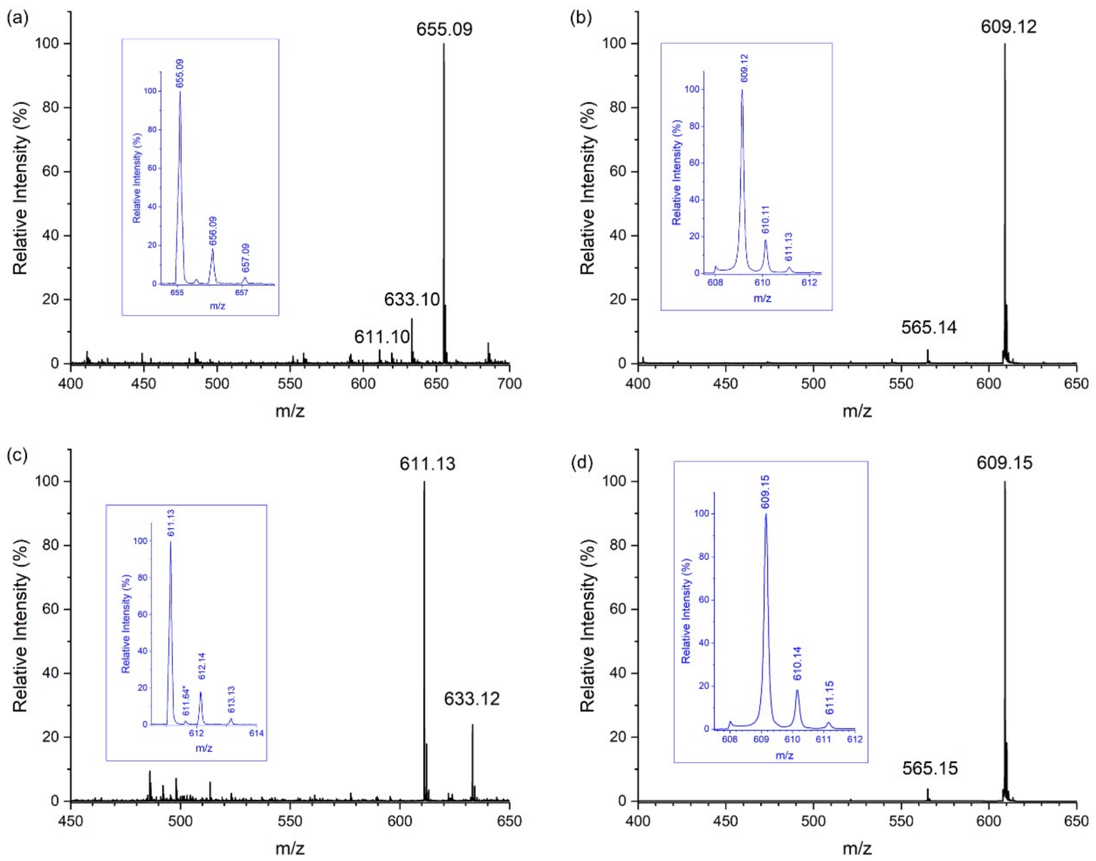

Figure S13. ESI mass spectra recorded in negative mode of samples containing acetonitrile: water (9:1). Samples were diluted from aqueous solutions containing Na[Bi(DOTA)·4H_2O (0.33 mM) incubated with 2 molar equivalents of M^{3+}(0.67 mMol) (M=Sb and Y) for at least 2 hours at 298 K. The ESI mass spectra of the incubation with $Sb_2(SO_4)_3$ recorded in **(a)** positive and **(b)** negative modes did not result in any substitution of the Bi^{3+} in $[Bi(DOTA)]^-$. The ESI mass spectra of the incubation with $Y(NO_3)_3{\cdot}6H_2O$ recorded in **(c)** positive and **(d)** negative modes did not result in any substitution of the Bi^{3+} in $[Bi(DOTA)]^-$. Only peaks corresponding to $[H_2Bi(DOTA)]^+$ (m/z 655.09) (expanded in inset), $[NaHBi(DOTA)]^+$ (m/z 633.12), and $[Na_2Bi(DOTA)]^+$ (m/z 535.03) were observed in ESI positive mass spectra, and $[Bi(DOTA)]^-$ (m/z 609.15) (expanded in inset) and $\{[Bi(DOTA)]\text{-}CO_2\}^-$ (m/z 565.15) were observed in ESI negative spectra.

2. PXRD

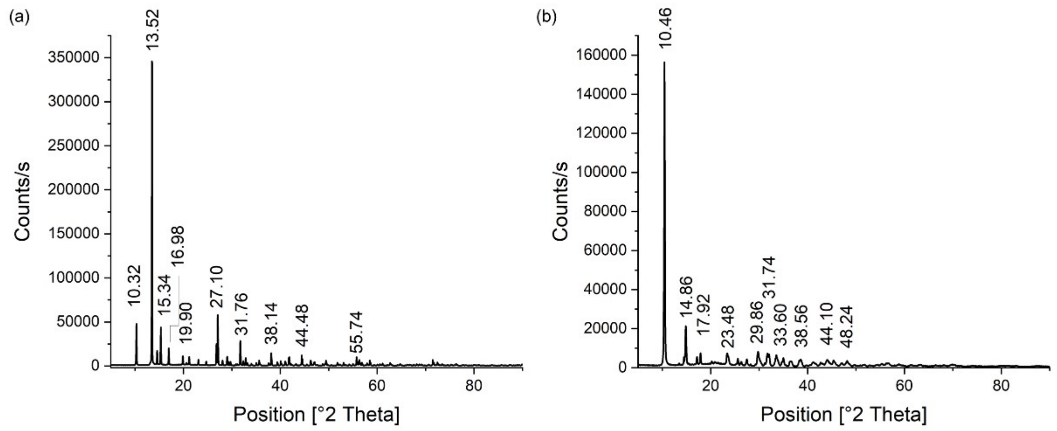

Figure S14. PXRD of (a) $[H_3O][Bi(DOTA)]H_2O$ and (b) $Na[Bi(DOTA)\cdot 4H_2O$

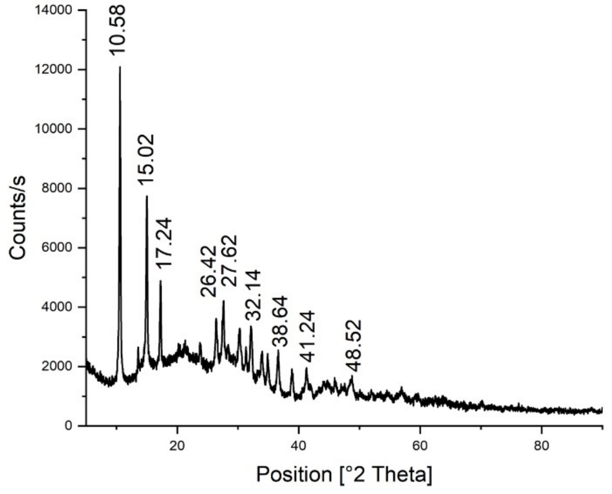

Figure S15. PXRD of $Na[Sb(DOTA)\cdot 4H_2O$

3. Crystallography

[H_6DOTA]Cl_2·4H_2O·DMSO:

Refinement

The crystal was twinned by rotation of 1.56° around [0.10 -0.99 0.09] (reciprocal) or [0.34 -0.90 0.27] (direct) and was refined using HKLF 5 data; the ratio of the components was 0.75035:0.24965. One of the chloride ions and one of the water molecules were disordered they were refined with Cl:H_2O occupancies of 9:1 at one site and 1:9 at the other. The minor components of the disorder were refined isotropically. DFIX constraints were applied to the O–H distances.

Further Figures

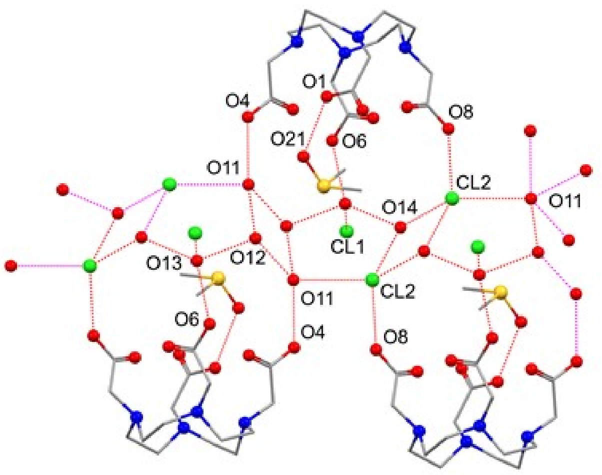

Figure S16. H-bonding network in [H_6DOTA]Cl_2·4H_2O·DMSO.

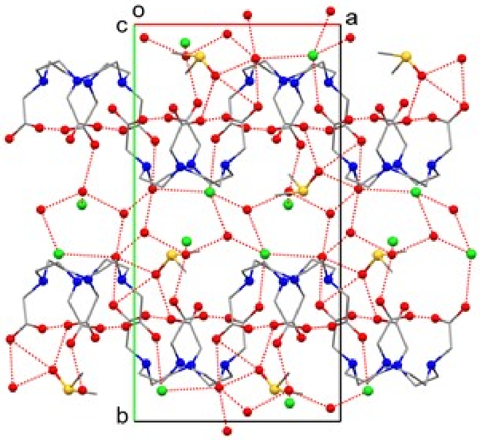

Figure S17. Packing diagram for [H_6DOTA]Cl_2·4H_2O·DMSO viewed down the *c* axis

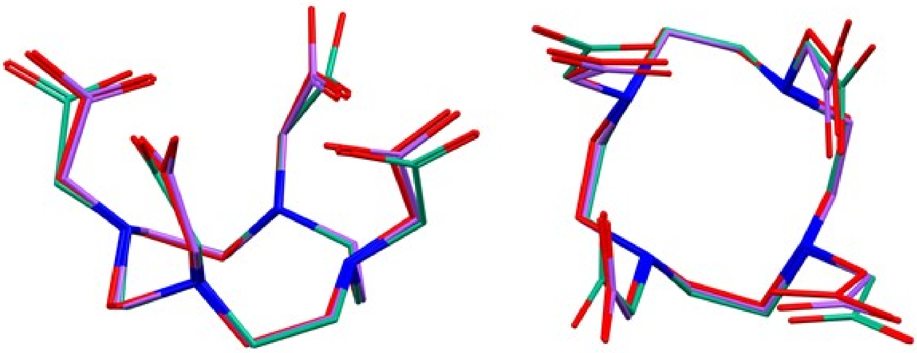

Figure S18. Overlays of the H_4DOTA or H_6DOTA^{2+} moieties from $[H_6DOTA]Cl_2·4H_2O·DMSO$ (carbon atoms shown red), $H_4DOTA.2H_2O$ ULEVAJ (green) and $H_6DOTA)Cl_2.5H_2O$ GOYBUR (purple).

Table S1. Hydrogen-bond geometry (Å, °) for $[H_6DOTA]Cl_2·4H_2O·DMSO$

D—H···*A*	*D*—H	H···*A*	*D*···*A*	*D*—H···*A*
O1—H1···O21	0.82	1.73	2.538 (3)	166
O4—H4···O11	0.79	1.90	2.675 (3)	167
O4—H4···Cl2′	0.79	2.12	2.860 (9)	157
O6—H6···O13	0.81	1.84	2.619 (2)	162
O8—H8···Cl2	0.78 (2)	2.27 (3)	2.991 (2)	154 (3)
O8—H8···O11′	0.78 (2)	2.04 (4)	2.79 (3)	163 (4)
N2—H2···N1	0.84 (3)	2.54 (3)	2.964 (3)	112 (2)
N2—H2···N3	0.84 (3)	2.55 (3)	2.957 (2)	111 (2)
N4—H4*A*···N3	0.86 (3)	2.46 (3)	2.928 (3)	115 (2)
C1—H1*A*···O1	0.97	2.54	3.034 (3)	112
C2—H2*A*···Cl1^{i}	0.97	2.77	3.727 (2)	169
C3—H3*B*···Cl2′^{ii}	0.97	2.72	3.599 (9)	150
C5—H5*A*···O2^{iii}	0.97	2.36	3.253 (3)	153
C5—H5*B*···Cl1^{iv}	0.97	2.71	3.566 (2)	148
C6—H6*A*···Cl1^{i}	0.97	2.82	3.783 (2)	174
C9—H9*A*···O5	0.97	2.63	3.132 (3)	113
C10—H10*A*···Cl1^{i}	0.97	2.77	3.732 (2)	172
C11—H11*A*···O3	0.97	2.63	3.124 (3)	112
C13—H13*A*···O6^{ii}	0.97	2.49	3.396 (3)	156
C13—H13*A*···O7	0.97	2.64	3.125 (3)	111

C13—H13*B*···Cl1^{ii}	0.97	2.76	3.595 (2)	144
C14—H14*A*···Cl1^{i}	0.97	2.73	3.684 (2)	170
C14—H14*B*···O12^{ii}	0.97	2.59	3.475 (3)	152
C15—H15*A*···O21^{v}	0.97	2.61	3.371 (3)	135
C22—H22*A*···Cl1^{vi}	0.96	2.76	3.688 (3)	164
C22—H22*B*···O14^{vii}	0.96	2.57	3.461 (4)	154
C22—H22*C*···O2	0.96	2.56	3.289 (4)	133
C21—H21*A*···O7	0.96	2.58	3.496 (4)	160
C21—H21*C*···O3	0.96	2.57	3.275 (3)	131
O12—H12*A*···O13	0.78 (2)	2.05 (2)	2.819 (3)	169 (4)
O12—H12*B*···O11^{viii}	0.77 (2)	2.24 (3)	2.956 (3)	155 (4)
O12—H12*B*···Cl2′^{viii}	0.77 (2)	2.43 (3)	3.102 (9)	148 (4)
O14—H14*C*···Cl2	0.82 (2)	2.33 (2)	3.117 (2)	164 (4)
O14—H14*C*···O11′	0.82 (2)	2.22 (4)	3.02 (3)	168 (4)
O14—H14*D*···Cl2^{vii}	0.81 (2)	2.32 (3)	3.092 (2)	159 (4)
O14—H14*D*···O11′^{vii}	0.81 (2)	2.31 (4)	3.12 (3)	171 (4)
O13—H13*C*···Cl1	0.80 (2)	2.24 (2)	3.0276 (17)	169 (3)
O13—H13*D*···O14	0.79 (2)	1.90 (2)	2.685 (3)	175 (4)
O11—H11*C*···O12	0.83 (2)	1.96 (2)	2.792 (3)	175 (4)
O11—H11*D*···Cl2^{ix}	0.82 (2)	2.31 (2)	3.104 (3)	162 (4)
O11′—H11*E*···O14^{vii}	0.83	2.46	3.12 (3)	137
O11′—H11*F*···Cl2′^{x}	0.84	2.53	3.16 (3)	132

Symmetry codes: (i) -*x*+3/2, *y*-1/2, -*z*+3/2; (ii) *x*-1/2, -*y*+1/2, *z*-1/2; (iii) *x*+1/2, -*y*+1/2, *z*+1/2; (iv) *x*+1/2, -*y*+1/2, *z*-1/2; (v) *x*-1/2, -*y*+1/2, *z*+1/2; (vi) *x*, *y*, *z*-1; (vii) -*x*+1, -*y*+1, -*z*+1; (viii) -*x*+2, -*y*+1, -*z*+1; (ix) *x*+1, *y*, *z*; (x) *x*-1, *y*, *z*.

Na[Sb(DOTA)]·4H2O and Na[Bi(DOTA)]·4H2O:

Refinement

These two isomorphous structures were treated identically. The unit cell dimensions for Na[Bi(DOTA)]·4H-$_2$O are the same as those reported previously (HADRAG) but the published structure was solved in C2/c with disorder of the sodium ion and several water molecules. This solution can be replicated with the current data sets. However, examination of the data showed that, while the data with *h*+*k*=odd are weak, they are not absent (~30% of the mean intensity for the data set, see below). Since the refinement in P2/c shows no disorder, we conclude that this is the correct choice and that the apparent centering is a consequence of the majority of the electron density being on the heavy atoms which have higher symmetry than the overall structure.

Table S2. Lattice type analyses for Na[Sb(DOTA)]·4H_2O and Na[Bi(DOTA)]·4H_2O.

Na[Sb(DOTA)]·4H_2O

Lattice exceptions:	P	A	B	C	I	F	Obv	Rev	All
N (total) =	0	48202	48187	48219	48202	72304	64217	64269	96342
N (int>3sigma) =	0	35850	37131	32267	37596	52624	49649	49666	74509
Mean intensity =	0.0	30.0	30.3	9.4	24.7	23.2	31.6	31.2	31.3
Mean int/sigma =	0.0	14.0	14.4	7.6	13.1	12.0	14.6	14.5	14.5

Lattice type: P chosen Volume: 2158.62

Na[Bi(DOTA)]·4H_2O:

Lattice exceptions:	P	A	B	C	I	F	Obv	Rev	All
N (total) =	0	48774	48895	48849	48825	73259	65123	65216	97738
N (int>3sigma) =	0	36257	36943	31610	38890	52405	50372	50676	75706
Mean intensity =	0.0	49.3	49.3	13.0	49.9	37.2	50.1	50.0	49.9
Mean int/sigma =	0.0	13.2	13.4	7.3	14.5	11.3	13.5	13.5	13.5

Lattice type: P chosen Volume: 2183.21

DFIX constraints were applied to the O–H distances of the coordinated water molecules. The Na1 – O11 bond is significantly longer than the others and this resulted in an unusual orientation of the (located) H atoms. Some additional DANG restraints were employed. The geometry could be further "improved" but this results in a poorer fit to the electron density.

Further Figures

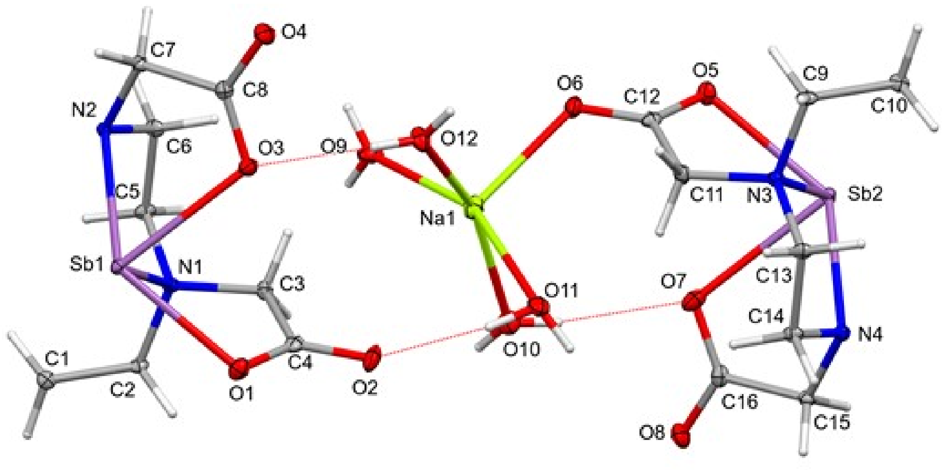

Figure S19. Asymmetric unit of Na[Sb(DOTA)]·4H_2O, showing atom labels and 50% probability ellipsoids, OH·····O H-bonds shown dotted.

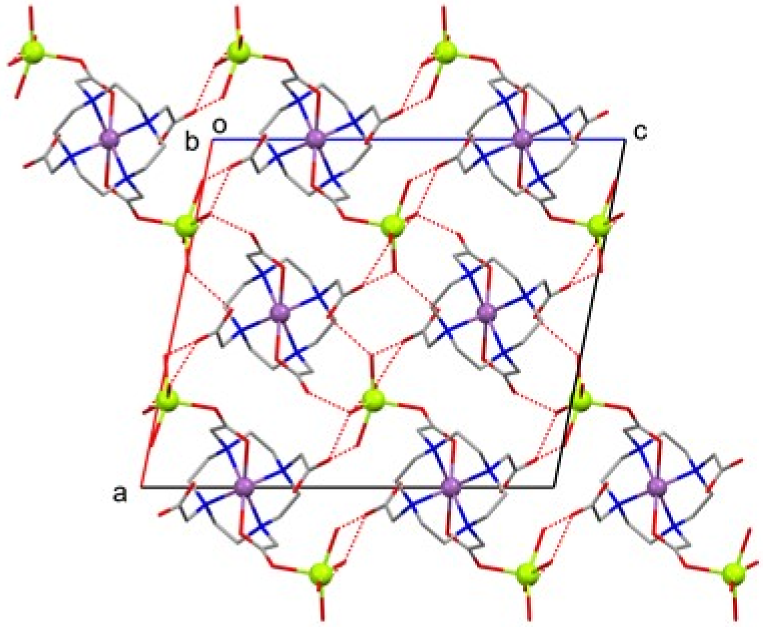

Figure S20. Packing diagram of Na[Sb(DOTA)]·4H_2O viewed down the *b* axis, showing OH---O H bonds as dotted lines, H atoms omitted for clarity.

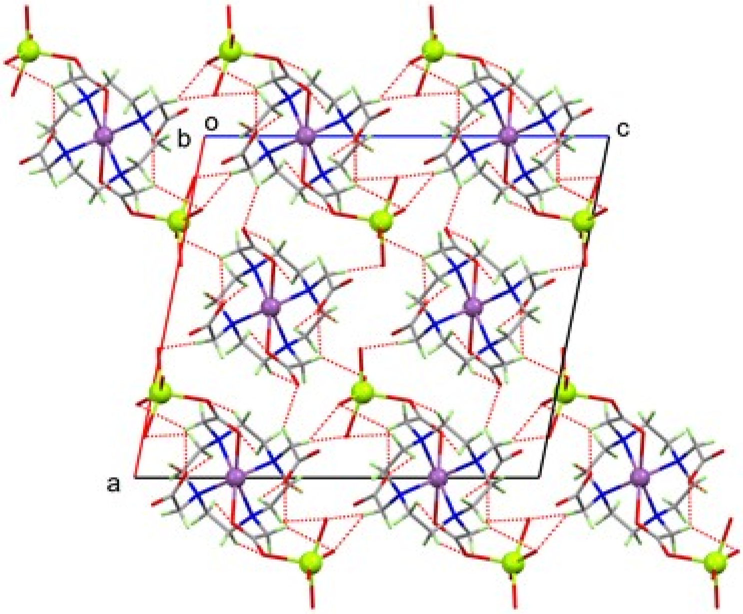

Figure S21. Packing diagram of Na[Sb(DOTA)]·$4H_2O$ viewed down the I axis, showing CH---O H bonds as dotted lines, H atoms of water molecules omitted for clarity.

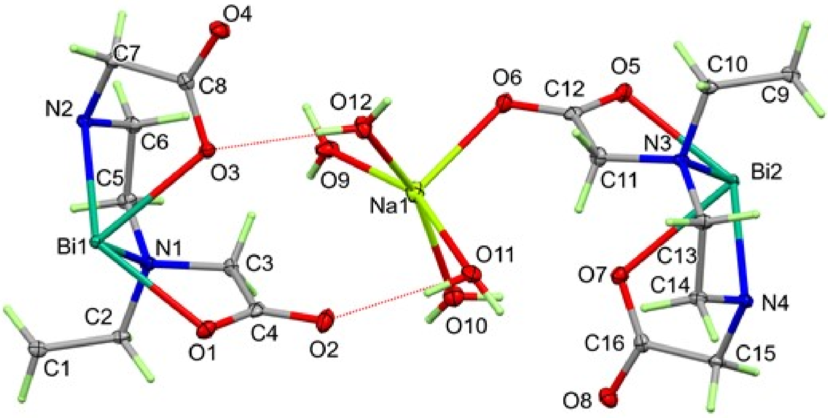

Figure S22. Asymmetric unit of Na[Bi(DOTA)]·$4H_2O$, showing atom labels and 50% probability ellipsoids, OH····O H-bonds shown dotted.

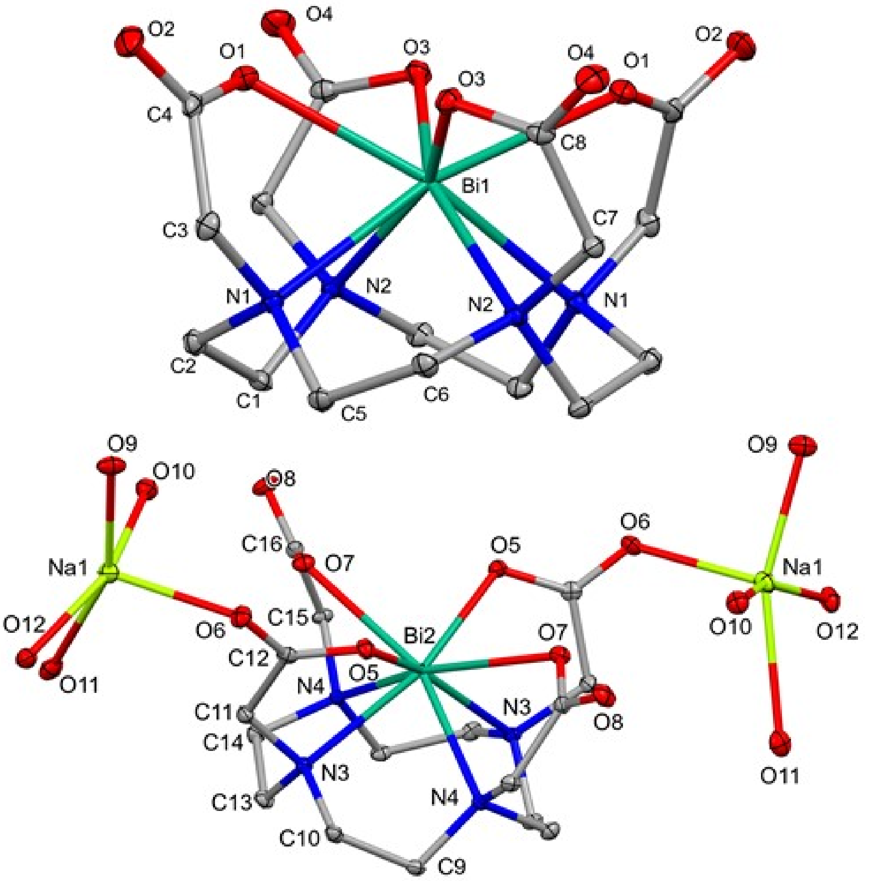

Figure S23. [Bi(DOTA)]$^-$ (top) and [(Na(H_2O)$_4$)$_2$Bi(DOTA)]$^+$(bottom) drawn with 50% probability ellipsoids. Carbon atoms are labelled for one asymmetric unit and H atoms are omitted.

Table S3. Hydrogen-bond geometry (Å, °) for Na[Sb(DOTA)]·4H_2O

D—H···*A*	*D*—H	H···*A*	*D*···*A*	*D*—H···*A*
O9—H9*C*···O4^{iv}	0.80 (1)	1.98 (2)	2.7782 (15)	176 (2)
O9—H9*D*···O11^{iii}	0.82 (2)	1.93 (2)	2.7451 (16)	177 (2)
O10—H10*C*···O7	0.80 (1)	2.20 (1)	2.9851 (16)	169 (2)
O10—H10*D*···O8^{vii}	0.81 (1)	2.06 (2)	2.8736 (16)	175 (2)
O11—H11*C*···O8^{vi}	0.82 (1)	2.01 (1)	2.8143 (16)	171 (2)
O11—H11*D*···O2	0.84 (1)	1.94 (2)	2.7556 (15)	164 (2)
O12—H12*A*···O3	0.80 (1)	2.10 (1)	2.8921 (15)	174 (2)
O12—H12*B*···O4^{viii}	0.80 (1)	2.05 (2)	2.8389 (15)	174 (2)
C1—H1*A*···O1^{iii}	0.97	2.59	3.2151 (17)	122
C2—H2*B*···O2^{iii}	0.97	2.59	3.5082 (18)	158
C3—H3*A*···O9	0.97	2.57	3.2284 (18)	125
C5—H5*A*···O3^{iii}	0.97	2.64	3.2465 (17)	121
C6—H6*A*···O3	0.97	2.61	3.0851 (17)	111
C7—H7*A*···O1^{i}	0.97	2.62	3.1186 (17)	112
C7—H7*B*···O12^{iv}	0.97	2.60	3.5453 (18)	164
C9—H9*A*···O6^{v}	0.97	2.62	3.5331 (17)	158
C10—H10*B*···O5^{v}	0.97	2.60	3.2315 (17)	123
C11—H11*B*···O7	0.97	2.62	3.1389 (18)	114
C11—H11*B*···O11	0.97	2.66	3.3417 (18)	128
C13—H13*A*···O10^{v}	0.97	2.63	3.3136 (17)	128
C14—H14*B*···O7	0.97	2.60	3.1004 (18)	112
C15—H15*A*···O10^{vi}	0.97	2.53	3.4763 (18)	166
C15—H15*A*···O11^{vi}	0.97	2.63	3.1655 (17)	115
C15—H15*B*···O2^{vi}	0.97	2.53	3.1362 (17)	121
C15—H15*B*···O5^{ii}	0.97	2.57	3.1129 (17)	116

Symmetry codes: (i) -*x*+1, *y*, -*z*+1/2; (ii) -*x*+2, *y*, -*z*+3/2; (iii) *x*, *y*+1, *z*; (iv) -*x*+1, -*y*+2, -*z*+1; (v) *x*, *y*-1, *z*; (vi) -*x*+2, -*y*+1, -*z*+1; (vii) -*x*+2, -*y*+2, -*z*+1; (viii) -*x*+1, -*y*+1, -*z*+1.

Table S4. Hydrogen-bond geometry (Å, °) for Na[Bi(DOTA)]·4H_2O

D—H···*A*	*D*—H	H···*A*	*D*···*A*	*D*—H···*A*
C1—H1*A*···O1^{iii}	0.99	2.62	3.2433 (18)	121
C3—H3*A*···O9	0.99	2.51	3.214 (2)	128
C6—H6*A*···O3	0.99	2.64	3.1199 (18)	110
C7—H7*B*···O12^{iv}	0.99	2.60	3.561 (2)	164
C9—H9*B*···O5^{v}	0.99	2.62	3.2524 (18)	122
C11—H11*B*···O11	0.99	2.61	3.335 (2)	130
C13—H13*A*···O10^{v}	0.99	2.64	3.3382 (19)	127
C14—H14*B*···O7	0.99	2.64	3.1330 (19)	111
C15—H15*A*···O10^{vi}	0.99	2.52	3.4918 (19)	166
C15—H15*A*···O11^{vi}	0.99	2.64	3.1627 (19)	113
C15—H15*B*···O2^{vi}	0.99	2.50	3.134 (2)	122
O9—H9*C*···O4^{iv}	0.83 (2)	1.95 (2)	2.7809 (17)	178 (2)
O9—H9*D*···O11^{iii}	0.85 (2)	1.89 (2)	2.7454 (17)	177 (2)
O10—H10*C*···O7	0.83 (2)	2.19 (2)	3.0072 (17)	167 (2)
O10—H10*D*···O8^{vii}	0.82 (2)	2.07 (2)	2.8885 (17)	175 (2)
O11—H11*D*···O2	0.845(15)	1.951(16)	2.7521(17)	157.9(19)
O11—H11*C*···O8^{vi}	0.83 (1)	1.99 (1)	2.8213 (17)	172 (2)
O12—H12*A*···O3	0.82 (2)	2.09 (2)	2.9075 (16)	173 (2)
O12—H12*B*···O4^{viii}	0.82 (2)	2.04 (2)	2.8514 (16)	174 (2)

Symmetry codes: (iii) *x*, *y*+1, *z*; (iv) -*x*+2, -*y*+2, -*z*+1; (v) *x*, *y*-1, *z*; (vi) -*x*+1, -*y*+1, -*z*+1; (vii) -*x*+1, -*y*+2, -*z*+1; (viii) -*x*+2, -*y*+1, -*z*+1.

$[H_3O][Bi(DOTA)]\cdot H_2O$

Refinement

The carboxylate arm is disordered and refined to occupancies of 76.5% for the major component and 23.5% for the minor component. The atoms of the minor component were refined isotropically.

The more difficult problem in this structure is the assignment of the remaining electron density and deriving a charge-balanced model. Assuming the Bi is in the +III state, charge balance requires one of the following: (a) one carboxylate group is not deprotonated; (b) there is one H_3O^+ per $[Bi(DOTA])^-$ ion; or (c) there is a Na^+ ion present, as in the $Na[Bi(DOTA)]\cdot 4H\text{-}_2O$ complex. Given the 4-fold symmetry and the disorder, it is not possible to detect whether or not there is a proton on the $DOTA^-$, though this seems not unreasonable at pH 2 and is also consistent with the 74:26 disorder of the carboxylate arms.

The region of electron density near the axis could be disordered water/H_3O^+ or disordered Na^+. The disorder means it is not clear from the O····X distances whether X is water/H_3O^+ or Na^+ – quite short H-bonds or quite long O····Na bonds are both possible. If this electron density is modelled as oxygen, disordered 50:50 on either side of the rotation-inversion axis (the coincident 2-fold rotation axis is more obvious), it will refine anisotropically and the residual electron density is low. Also, there are 3 small areas of electron density in approximately the right positions for 3 H atoms. Fitting this as 2 half-occupancy H_3O^+ ions would give $2[H_3O]^+$ per $[Bi(DOTA)]^-$ i.e. incorrect charge – so it has been modelled as ½(H_5O_2), i.e. one H_2O and one H_3O^+ per bismuth ion.

Replacing the H_3O^+/H_2O with 0.25 occupancy Na^+ (appropriate occupancy for charge balance) gave a less satisfactory model (R1 1.47% with higher residual electron density, and the Na does not refine anisotropically without severe restraints). The fit might perhaps be improved by disordering a partial-occupancy water molecule on top of the Na – but that seems unjustifiably complex.

So, on balance, $[H_3O][Bi(DOTA)]\cdot H_2O$ seems like the best model, with the caveat that the location of the acidic proton is not certain.

The packing diagram (Figure S25) is reminiscent of the packing for the $Na[Bi(DOTA)]\cdot 4H_2O$ complexes (Figure S23), with the H_3O^+/H_2O units playing a similar role to the hydrated sodium ion. Again, the $[Bi(DOTA)]^-$ ions are stacked into columns parallel to the c axis *via* CH····O hydrogen bonds, as shown in the main manuscript (Figure 6).

Further Figures

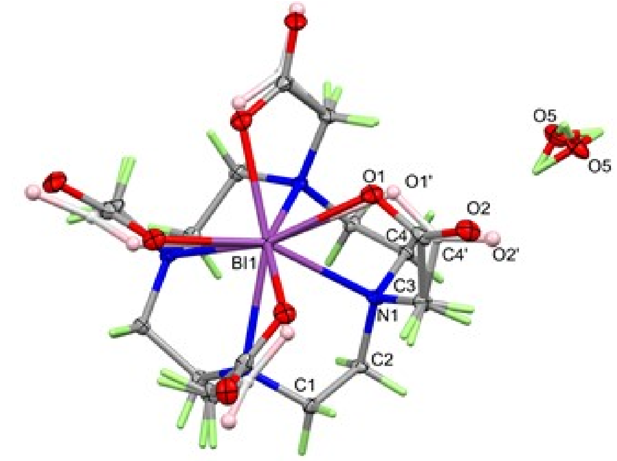

Figure S24. $[H_3O][Bi(DOTA)]\cdot H_2O$ showing disorder in the carboxylate groups and the H_3O+/H_2O **with 50%** probability ellipsoids

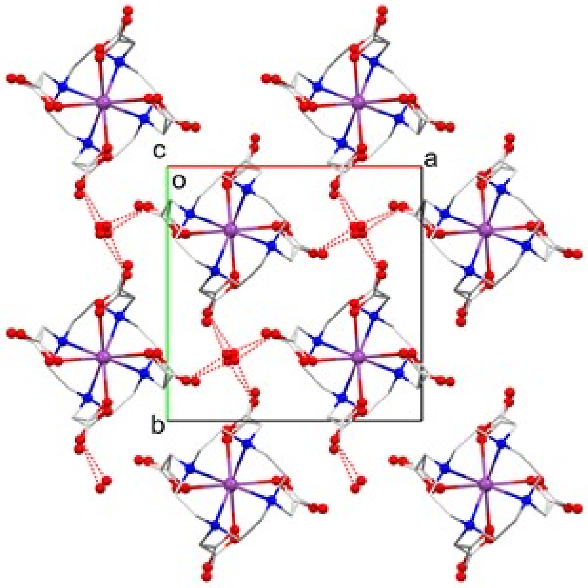

Figure S25. Packing diagram for $[H_3O][Bi(DOTA)]\cdot H_2O$ viewed down the *c* axis. Hydrogen bonds **shown as** dotted lines.

Table S5. Hydrogen-bond geometry (Å, °) for $[H_3O][Bi(DOTA)]\cdot H_2O$

D—H···*A*	*D*—H	H···*A*	*D*···*A*	*D*—H···*A*
C1—H1*A*···O2a^{iv}	0.99	2.65	3.589 (4)	159
C1—H1*B*···O2a^{v}	0.99	2.42	3.381 (2)	162
C1—H1*B*···O1′b	0.99	2.56	3.094 (7)	114
C1—H1*B*···O2′b^{v}	0.99	2.40	3.385 (5)	171
C2—H2*B*···O1a^{iv}	0.99	2.59	3.228 (2)	122
C3a—H3Aa···O5^{vi}	0.99	2.58	3.410 (6)	141
C3a—H3Ba···O2a^{vii}	0.99	2.60	3.575 (2)	167
C3′b—H3′Bb···O1′b^{iii}	0.99	2.58	3.302 (7)	129
O5—H5*A*···O5^{viii}	0.73 (2)	1.90 (2)	2.600 (4)	160 (1)
O5—H5*A*···O5^{ix}	0.73 (2)	1.90 (2)	2.600 (4)	160 (1)
O5—H5*B*···O2a	0.82 (2)	1.78 (2)	2.563 (8)	161 (3)
O5—H5*B*···O2′b	0.82 (2)	1.67 (2)	2.484 (9)	172 (3)

Symmetry codes: (iii) -*y*+3/2, *x*, *z*; (iv) *x*, *y*, *z*+1; (v) -*y*+1, *x*-1/2, -*z*+1; (vi) *y*+1/2, -*x*+1, -*z*+1; (vii) -*x*+2, -*y*+1, -*z*+1; (viii) *y*+1/2, -*x*+1, -*z*; (ix) -*y*+1, *x*-1/2, -*z*.

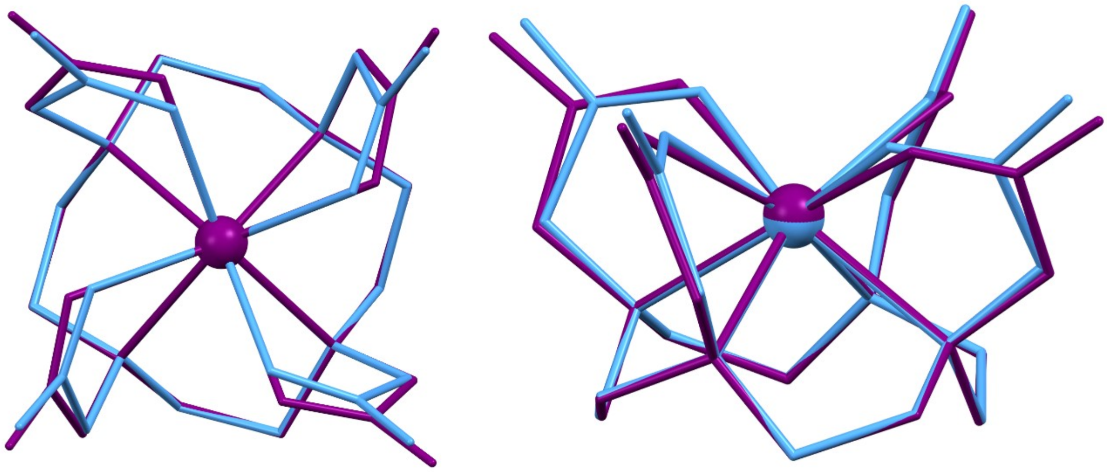

Figure S26. Overlay of the structures of $[Sc(DOTA)]^-$ (pale blue from JOGZEM) and $[Sb(DOTA)]^-$(purple) showing the different positions of the metal ions within the cavity. The four N atoms were used for the overlay mapping.

Table S6. Expanded version of table 1 with selected bond lengths (Å) for Na[Sb(DOTA)]·4H_2O and Na[Bi(DOTA)]·4H_2O, **[H_3O][Bi(dota)]·H_2O** and structures from literature containing $[Sc(DOTA)]^-$.[a]

Na[Sb(DOTA)]·4H-$_2$O		Na[Bi(DOTA)]·4H-$_2$O		[H_3O][Bi(dota)]·H_2O		3(Na[Sc(DOTA)]) ·NaOH·18H_2O[c]		K[Sc(dota)][H_6dota]Cl_2·4H_2 O[d]	
Sb1—O1	2.5011 (10)	Bi1—O1	2.4993 (11)	Bi1—O1	2.439 (3)	Sc1 – O1	2.151(2)	Sc1 – O51A	2.2228(15)
Sb1—O3	2.5971 (10)	Bi1—O3	2.5715 (11)			Sc2 – O3	2.147(2)		
Sb2—O5	2.5146 (10)	Bi2—O5	2.5012 (11)			Sc3 – O5	2.163(2)		
Sb2—O7	2.6929 (11)	Bi2—O7	2.6336 (11)						
Sb1—N1	2.4563 (11)	Bi1—N1	2.5288 (13)	Bi1—N1	2.5396 (11)	Sc1 – N1	2.441(2)	Sc1 – N1A	2.4100(17)
Sb1—N2	2.4453 (11)	Bi1—N2	2.5170 (12)			Sc2 – N2	2.446(3)		
Sb2—N3	2.4421 (12)	Bi2—N3	2.5256 (13)			Sc3 – N3	2.450(2)		
Sb2—N4	2.4277 (11)	Bi2—N4	2.5034 (12)						
M—N_4 plane[b]	1.340, 1.333		1.423, 1.429		1.450		1.327, 1.336, 1.330		1.300
M—O_4 plane[b]	1.155, 1.162		1.113, 1.106		1.116		1.007, 0.989, 1.019		1.172
Na1—O6	2.3483 (12)	Na1—O6	2.3531 (13)						
Na1—O9	2.3074 (13)	Na1—O9	2.3093 (13)						
Na1—O10	2.3653 (12)	Na1—O10	2.3705 (13)						
Na1—O11	2.6005 (13)	Na1—O11	2.6204 (14)						
Na1—O12	2.3341 (12)	Na1—O12	2.3383 (13)						

[a]In the case of disordered structures, values are quoted for the major component.

[b]Distance of the metal ion from the mean plane of the N_4 or O_4 donors.

[c] F. Benetollo, G. Bombieri, L. Calabi, S. Aime, M. Botta, *Inorg. Chem.* **2003**, 42, 148 (REFCODE: LUQCIJ) Note that the macrocyclic ring conformation in this complex is different from all the other complexes discussed in this paper.

[d] M. Pniok, V. Kubicek, J. Havlickova, J. Kotek, A. Sabatie-Gogova, J. Plutnar, S. Huclier-Markai, P. Hermann, *Chem. Eur. J.* **2014**, 20, 7944 (REFCODE: JOGZEM)
